# Supplementary material for: Unsupervised representation learning of chromatin images identifies changes in cell state and tissue organization in DCIS
Source: Nat Commun. 2024 Jul 20;15:6112. doi: 10.1038/s41467-024-50285-1 (PMC11271523; doi:10.1038/s41467-024-50285-1)
Supplement: Supplementary file 1 — Supplementary Information [file 41467_2024_50285_MOESM1_ESM.pdf]

Supplementary Information:
Unsupervised representation learning of chromatin images identifies changes in cell state and tissue organization in DCIS

Xinyi Zhang[1,2], Saradha Venkatachalapathy[3,4], Daniel Paysan[3,4], Paulina Schaerer[3,4], Claudio Tripodo[5,6], Caroline Uhler[1,2,*], GV Shivashankar[3,4,*]

[1] Massachusetts Institute of Technology, U.S.A.
[2] Broad Institute of MIT and Harvard, U.S.A.
[3] ETH Zurich, Switzerland
[4] Paul Scherrer Institute, Switzerland
[5] Tumor Immunology Unit, University of Palermo, Italy
[6] IFOM, FIRC Institute of Molecular Oncology, Milan, Italy

* To whom correspondence should be addressed; E-mail: cuhler@mit.edu, gshivasha@ethz.ch

This PDF file includes:

Supplementary Figures 1-25

Supplementary Data 1-2

Supplementary References

Nuclei segmentation

a Manual segmentation used for training

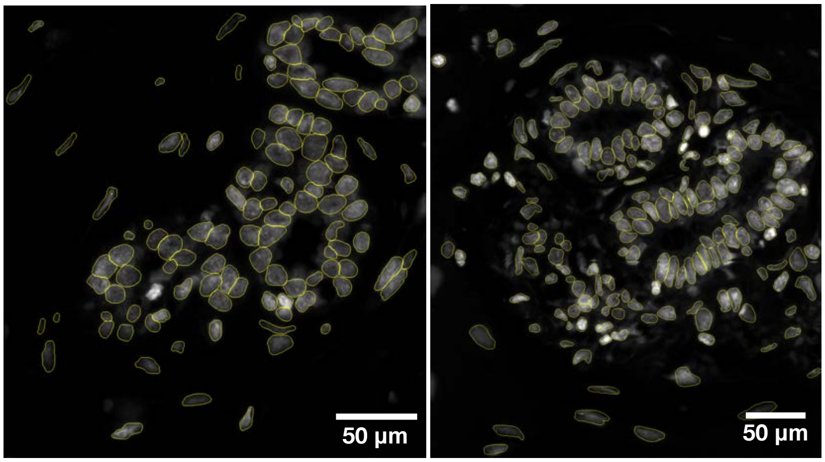

b Training and validation losses of the segmentation model

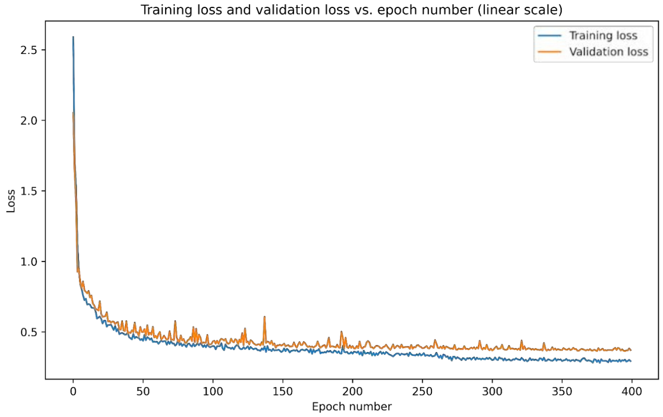

c Examples of segmentation

Breast tissue   Hyperplasia   IDC

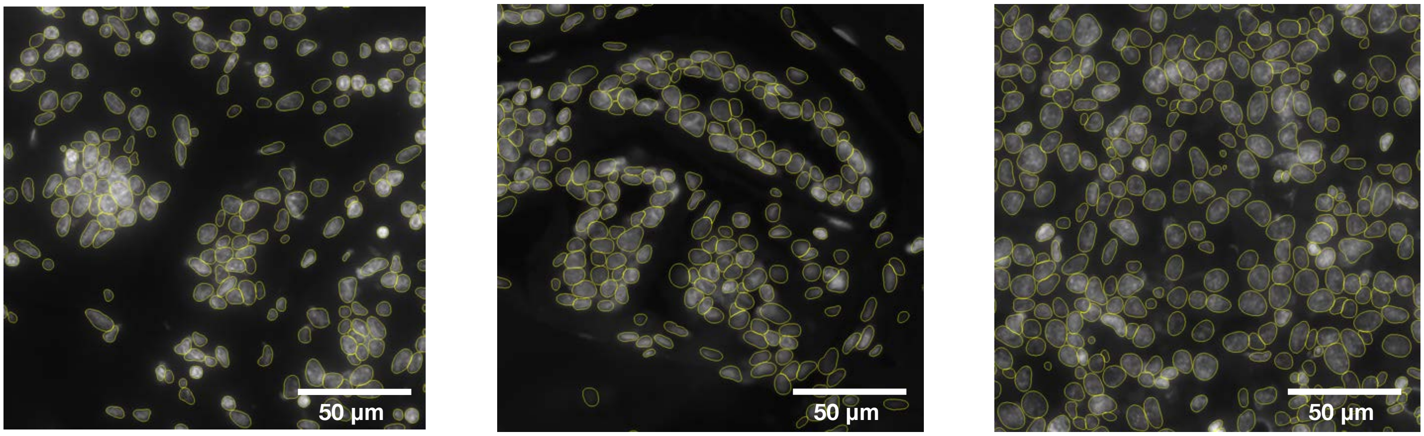

d Automatic duct segmentation through thresholding

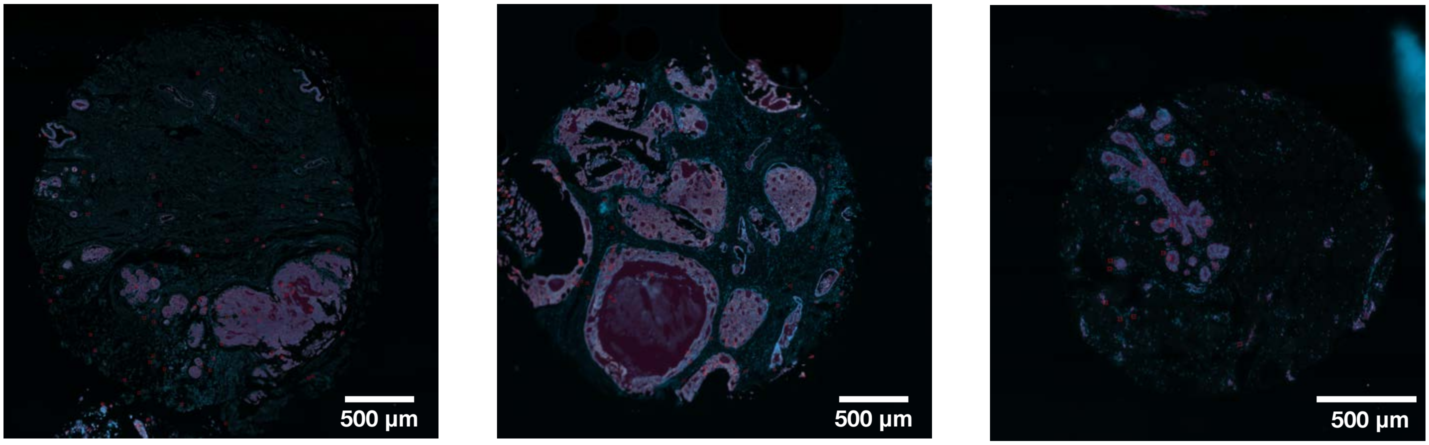

Supplementary Figure 1

Supplementary Figure 1. Image segmentation.

(a) Representative examples of manual segmentation of nuclei used for training the StarDist model.

(b) Training and validation losses of the StarDist model.

(c) Representative examples of nuclear segmentation using the trained StarDist Model.

(d) Representative examples of breast duct segmentation.

	Quality of cells without imaging artifact	Segmentation errors of cells without imaging artifact	If segmentation errors is related to disease stages	If imaging artifact is related to disease stage	Summary of imaging artifacts
**P0. Breast tissue**	Excellent (equivalent to a very accurate manual segmentation.)	Very rare non-segmented nuclei and segmented non-nuclear artifacts. Very fusate nuclei are less efficiently segmented.	No	NA	None
**P1. Cancer adjacent breast tissue**	Excellent (almost perfect when the stroma does not have background signal)	Very fusate nuclei are not recognized (fibroblasts)	No	NA	None
**P2. IDC (Breast tissue)**	Excellent	Very few nuclei with cleaved morphology are not properly segmented (spindle-shaped nuclei of stromal cells).	No (will not impact epithelial cells either normal or malignant)	NA	None
**P3. Hyperplasia**	Excellent	None	NA	NA	None
**P4. Atypical hyperplasia**	Excellent	Spindle-shaped nuclei of stromal cells less properly segmented as in P2.	NA	NA	None
**P5. DCIS and breast tissue**	Excellent	None	NA	No	In some regions, high DAPI background signal impairs proper segmentation.
**P6. DCIS**	Excellent	None	NA	No	In some regions, high DAPI background signal impairs proper segmentation.
**P7. DCIS with early infiltration**	Excellent (Crowding of the cells and the brightness of DAPI in one patch would not have permitted manual segmentation.)	None	NA	NA	None
**P8. Micropapillary DCIS with early infiltration**	Excellent	Few cancer nuclei in one patch are not properly segmented	No	NA	None
**P9. IDC and breast tissue**	Excellent	None	NA	NA	None
**P10. IDC**	Excellent	Few minor errors	No	NA	None

Pathologist’s summary of our automatic segmentation:
Excellent, equivalent to a very accurate manual segmentation.

Supplementary Figure 2

Supplementary Figure 2. Summary of the assessment of our nuclear segmentation by a pathologist.

Pathologist's assessment of our segmentation of normal breast tissue (P0)

The quality of the segmentation is excellent, it is equivalent to a very accurate manual segmentation. The only minor issue is the presence of very rare non-segmented nuclei (blue arrow) and segmented non-nuclear artifacts (yellow arrow). It seems that some very fusate (flat) nuclei are less efficiently segmented.

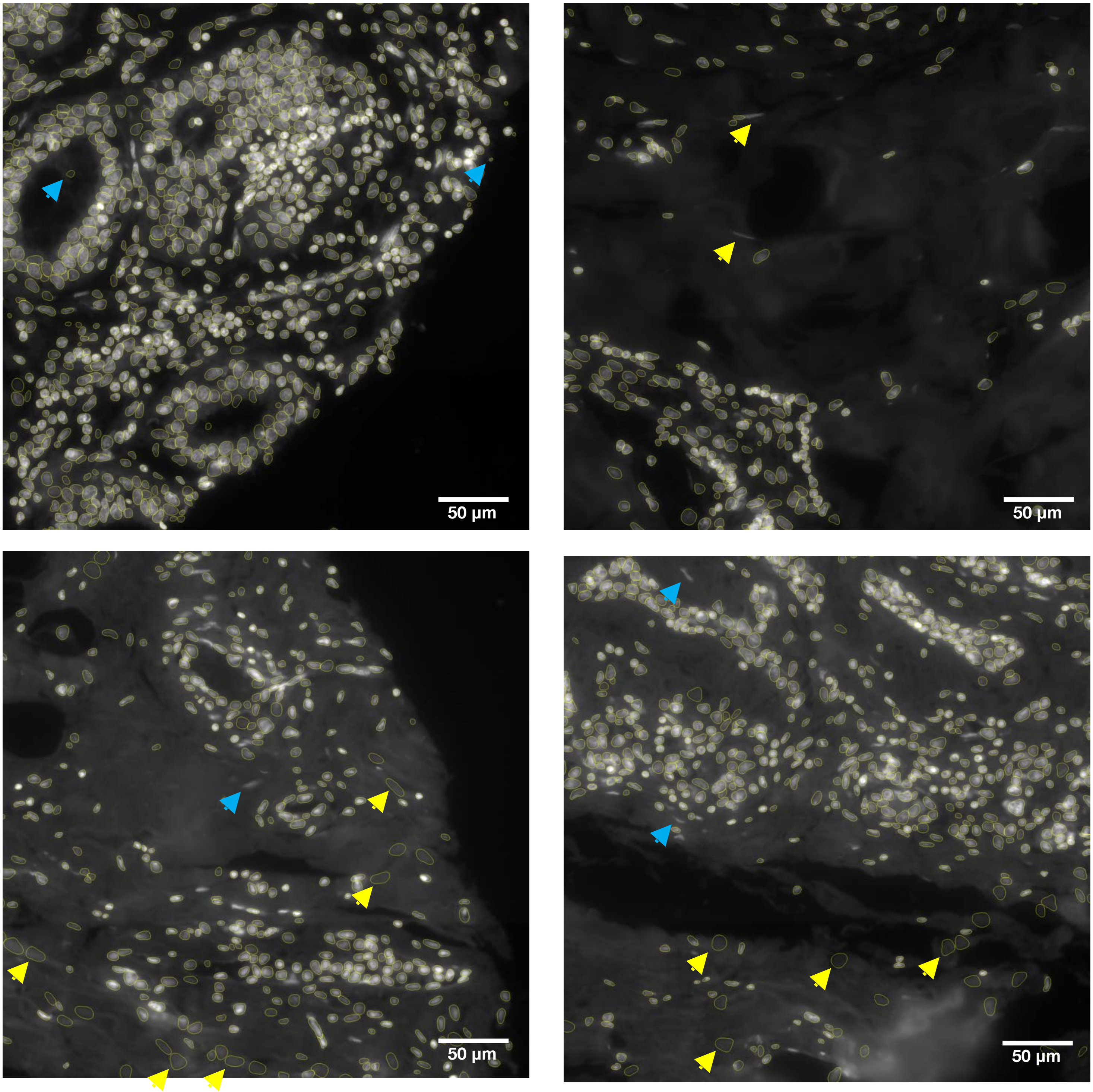

Supplementary Figure 3

Supplementary Figure 3. Examples of a pathologist's assessment of our nuclear segmentation of normal breast tissue samples.

Pathologist’s assessment of our segmentation of hyperplasia

Excellent segmentation.

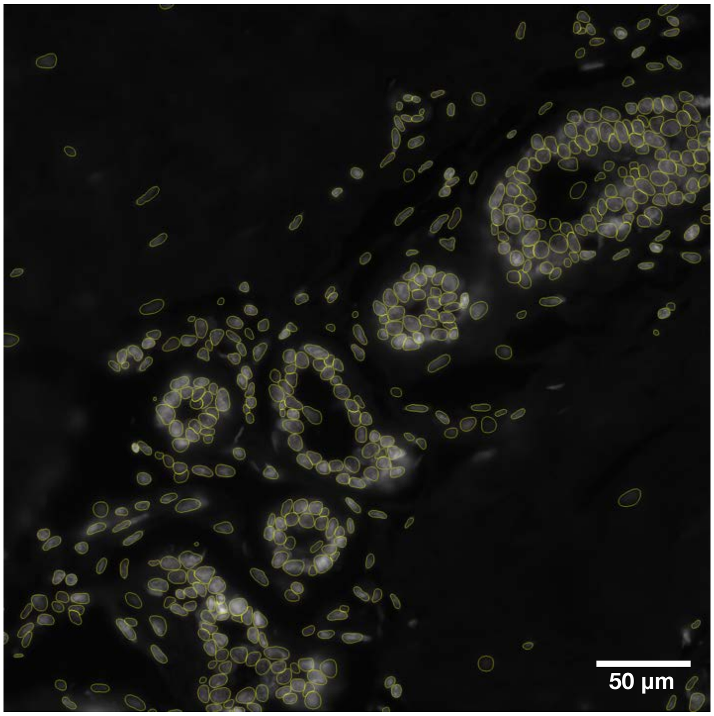

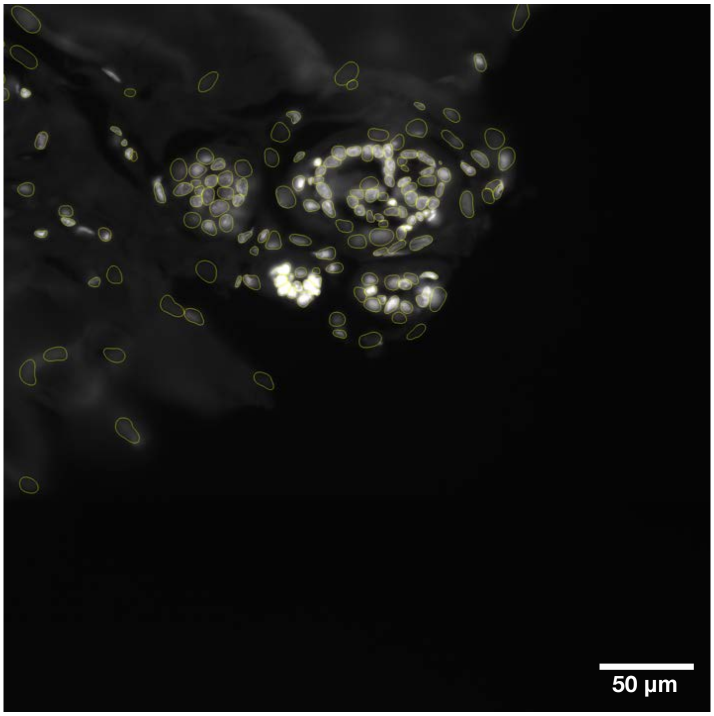

Pathologist’s assessment of our segmentation of atypical hyperplasia

These regions are excellently segmented. The same caveat applies for splindle-shaped nuclei of stromal cells: Some nuclei with cleaved morphology are not properly segmented, but they are very few.

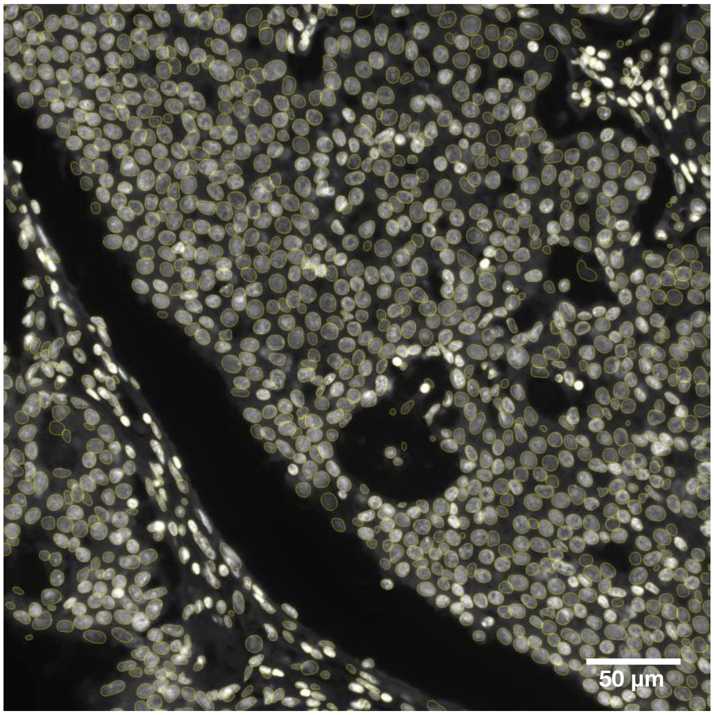

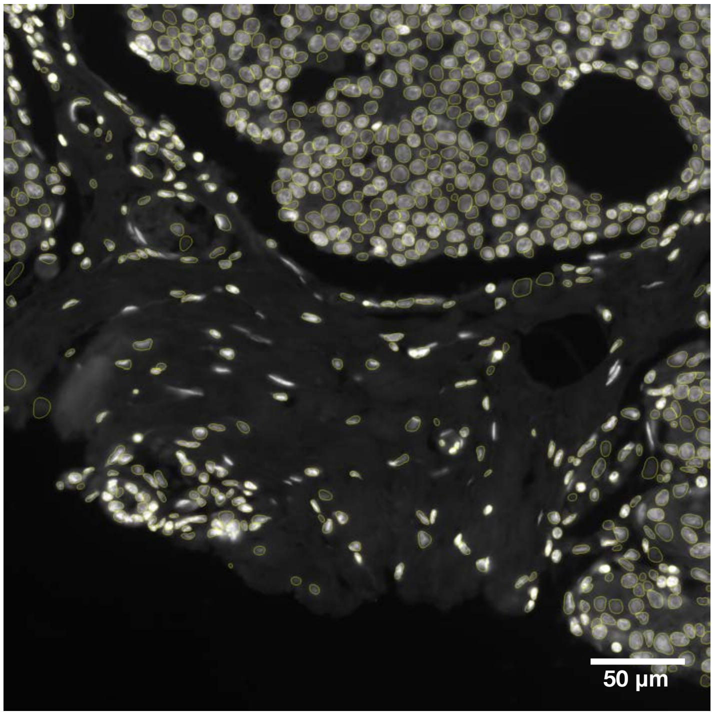

Supplementary Figure 4

Supplementary Figure 4. Examples of a pathologist's assessment of our nuclear segmentation of hyperplasia and atypical hyperplasia samples.

Pathologist’s assessment of our segmentation of DCIS

Excellent segmentation.

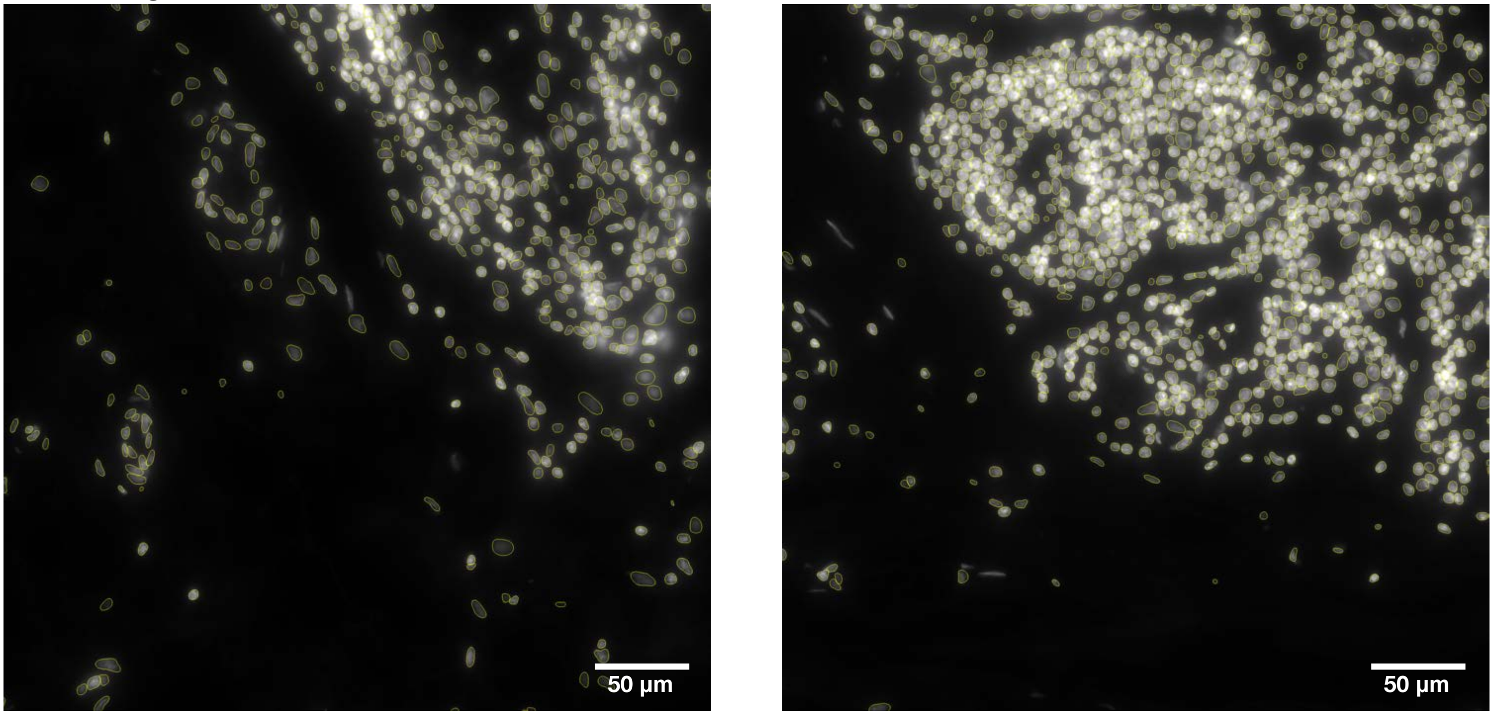

Pathologist’s assessment of our segmentation of DCIS with early infiltration

Excellent segmentation. This is a clear example where the crowding of the cells and the brightness of the DAPI would have not permitted manual segmentation in the focus highlighted on the right.

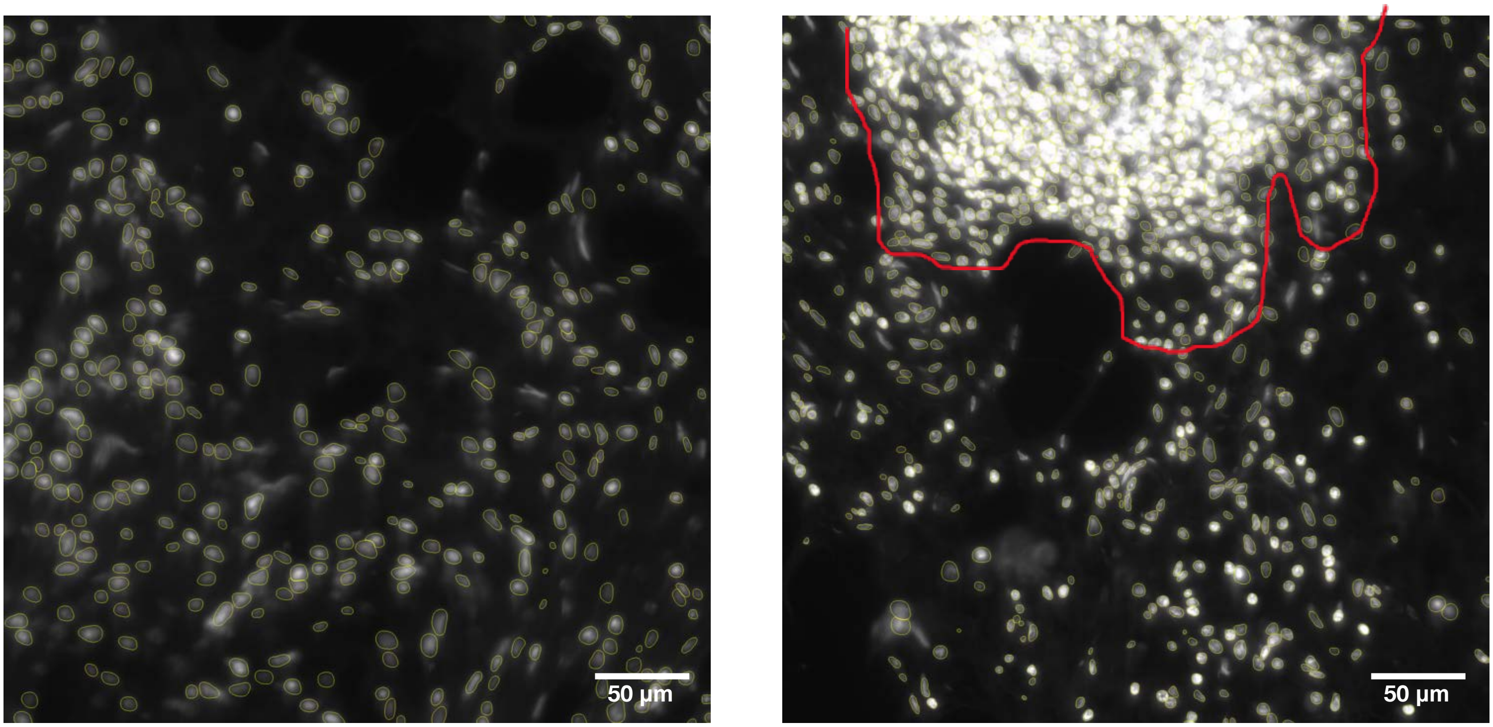

Supplementary Figure 5

Supplementary Figure 5. Examples of a pathologist's assessment of our nuclear segmentation of DCIS samples.

Pathologist's assessment of our segmentation of IDC

Excellent segmentation.

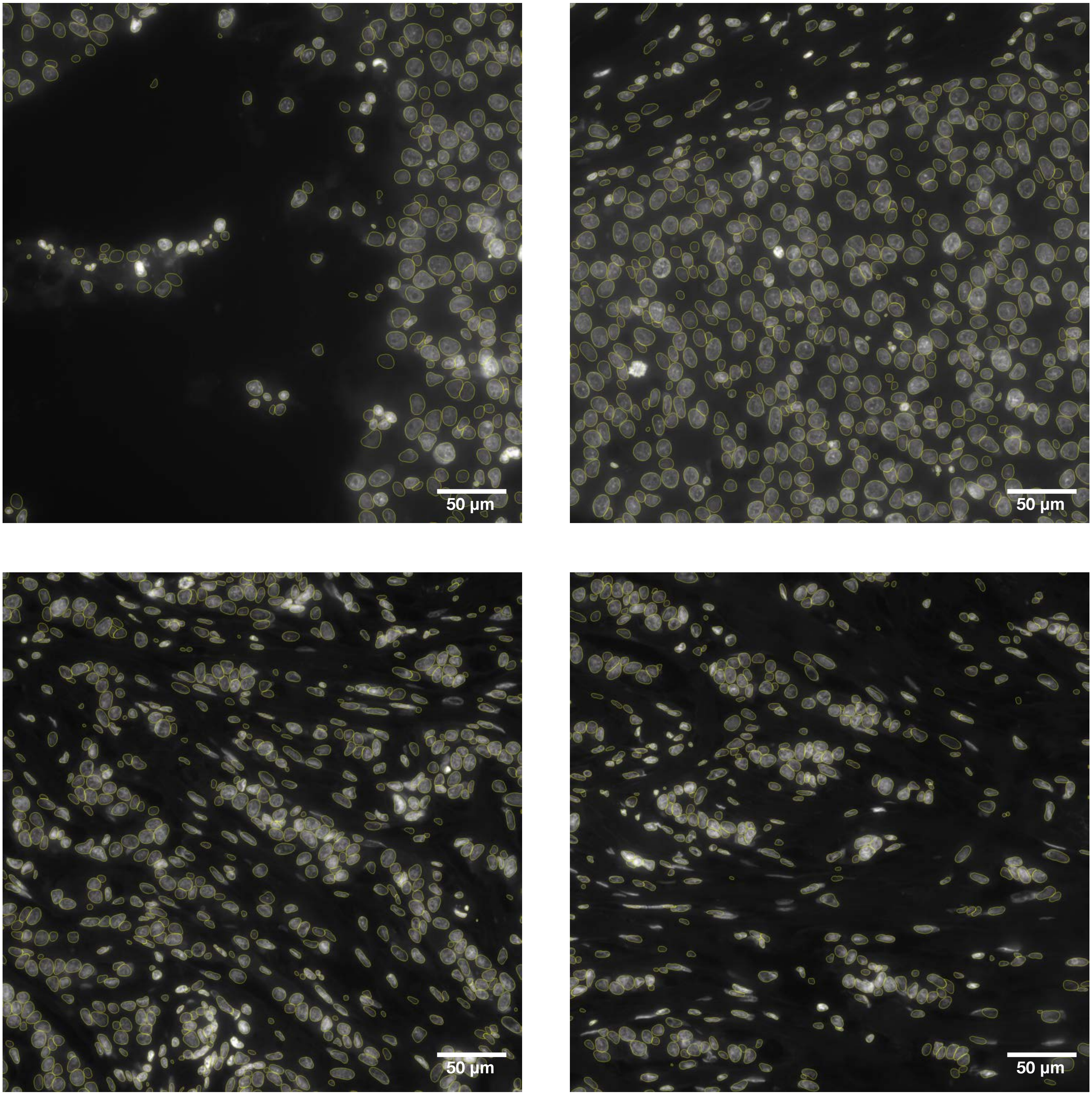

Supplementary Figure 6

Supplementary Figure 6. Examples of a pathologist's assessment of our nuclear segmentation of IDC samples.

Micropapilary DCIS with early infiltration

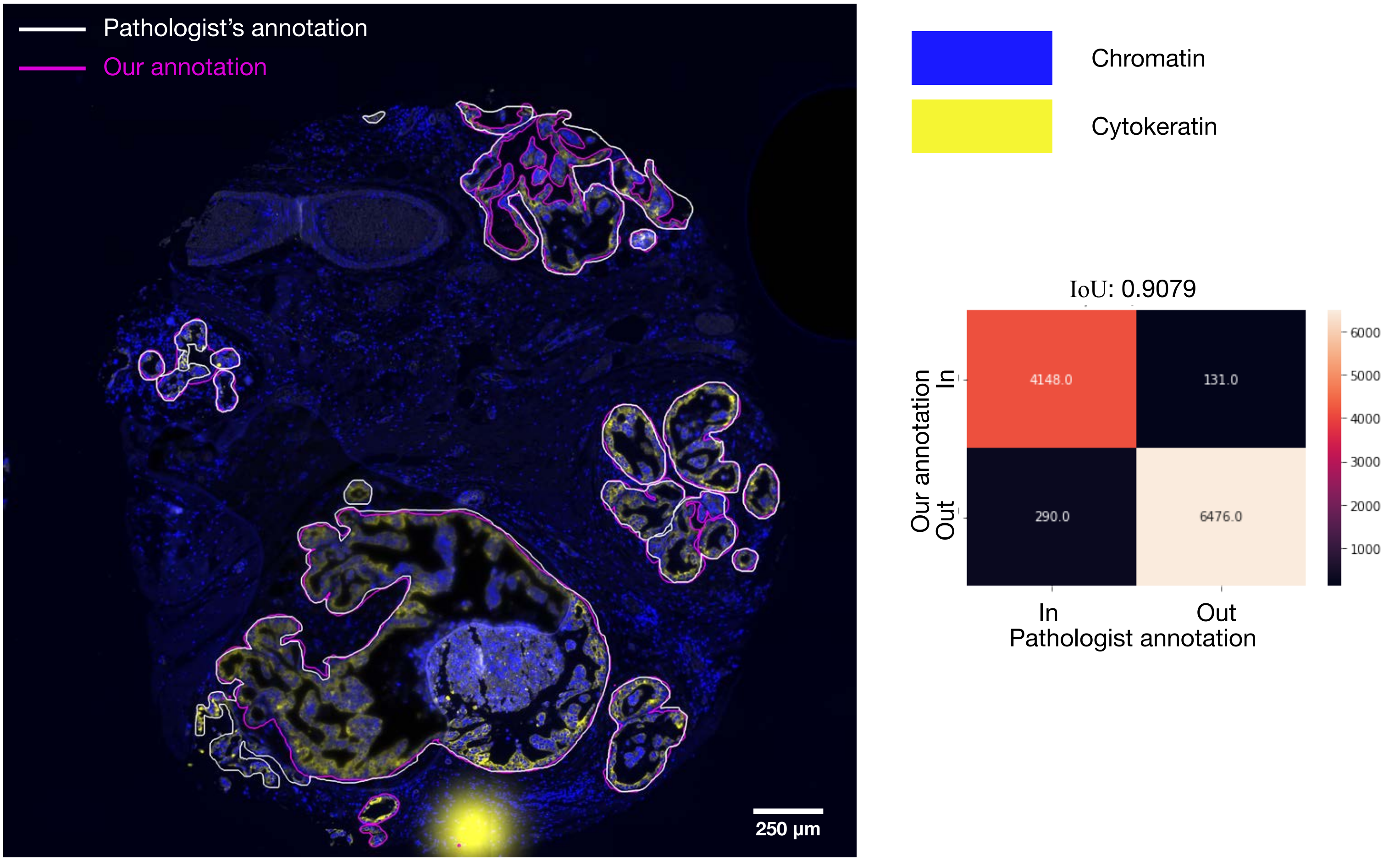

DCIS with early infiltration

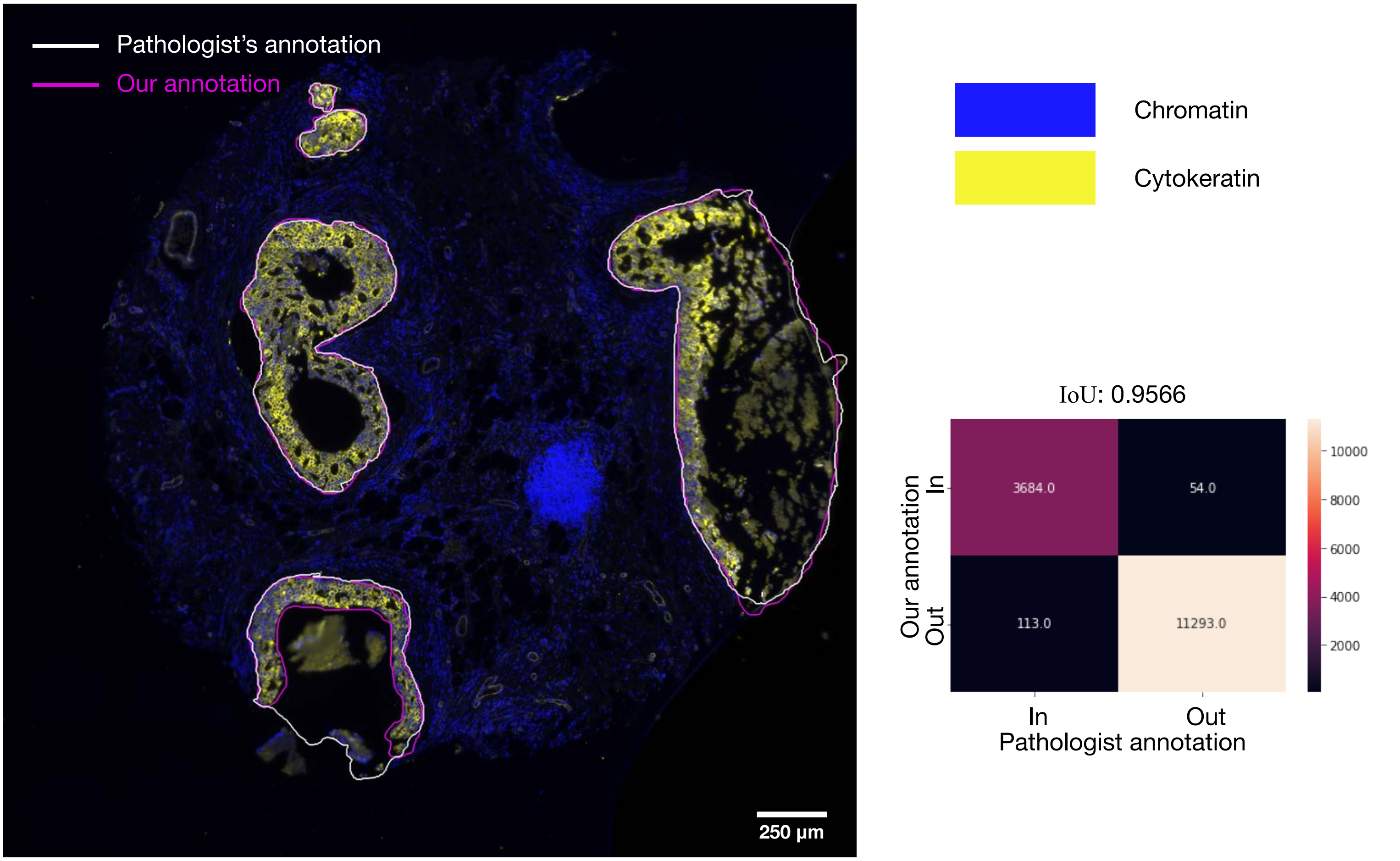

Supplementary Figure 7

Supplementary Figure 7. Comparison of our duct annotation to a pathologist's annotation in DCIS with early infiltration. Our annotation of ducts is outlined in pink and the pathologist's annotation is outlined in white. Heatmaps show the number of cells that are inside or outside of the ducts in our annotation compared to the number of cells in the pathologist's annotation. IoU is the intersection over union that computes the fraction of cells that are assigned with the same annotation by the two annotation sources compared to the total number of cells.

DCIS

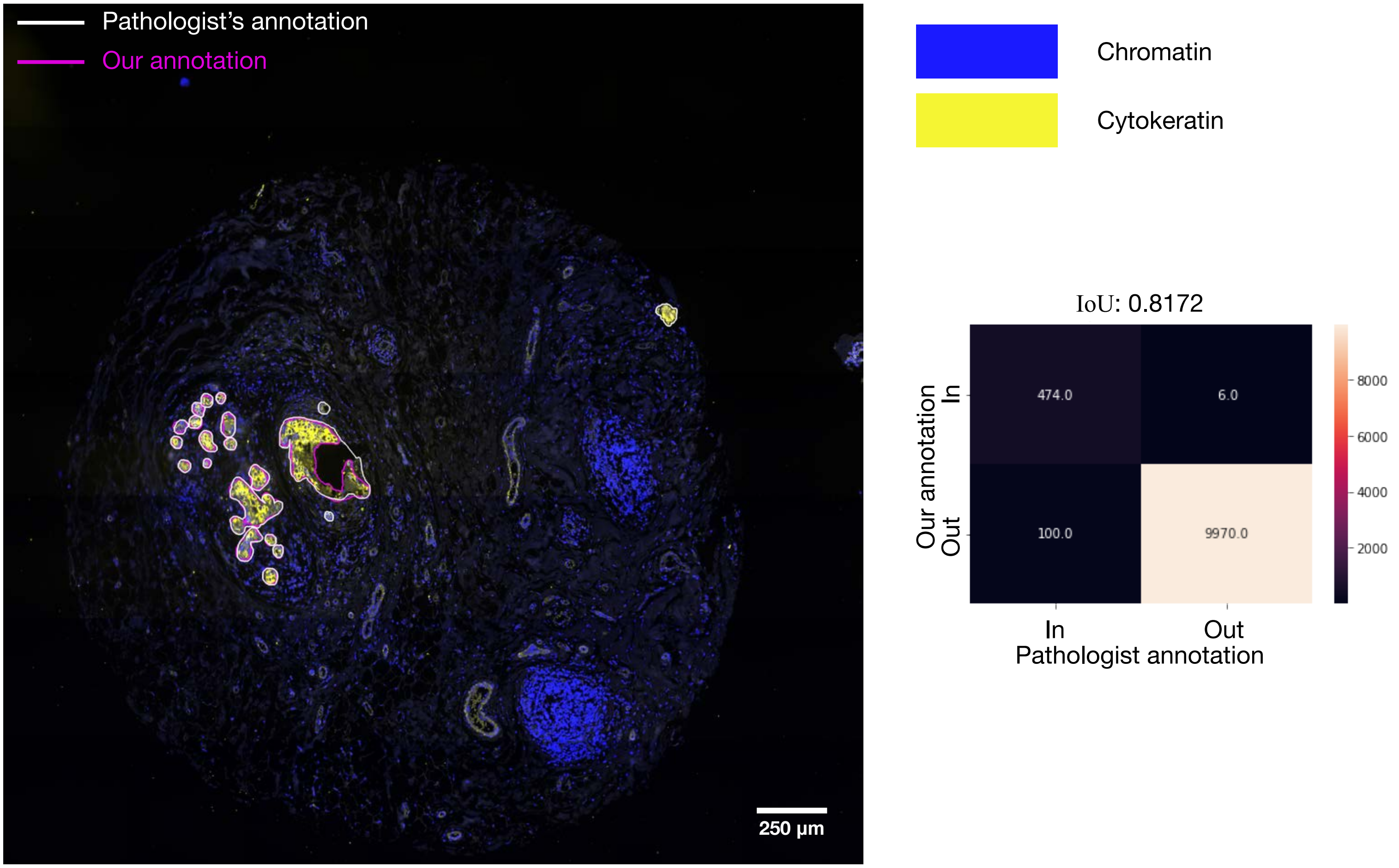

DCIS and breast tissue

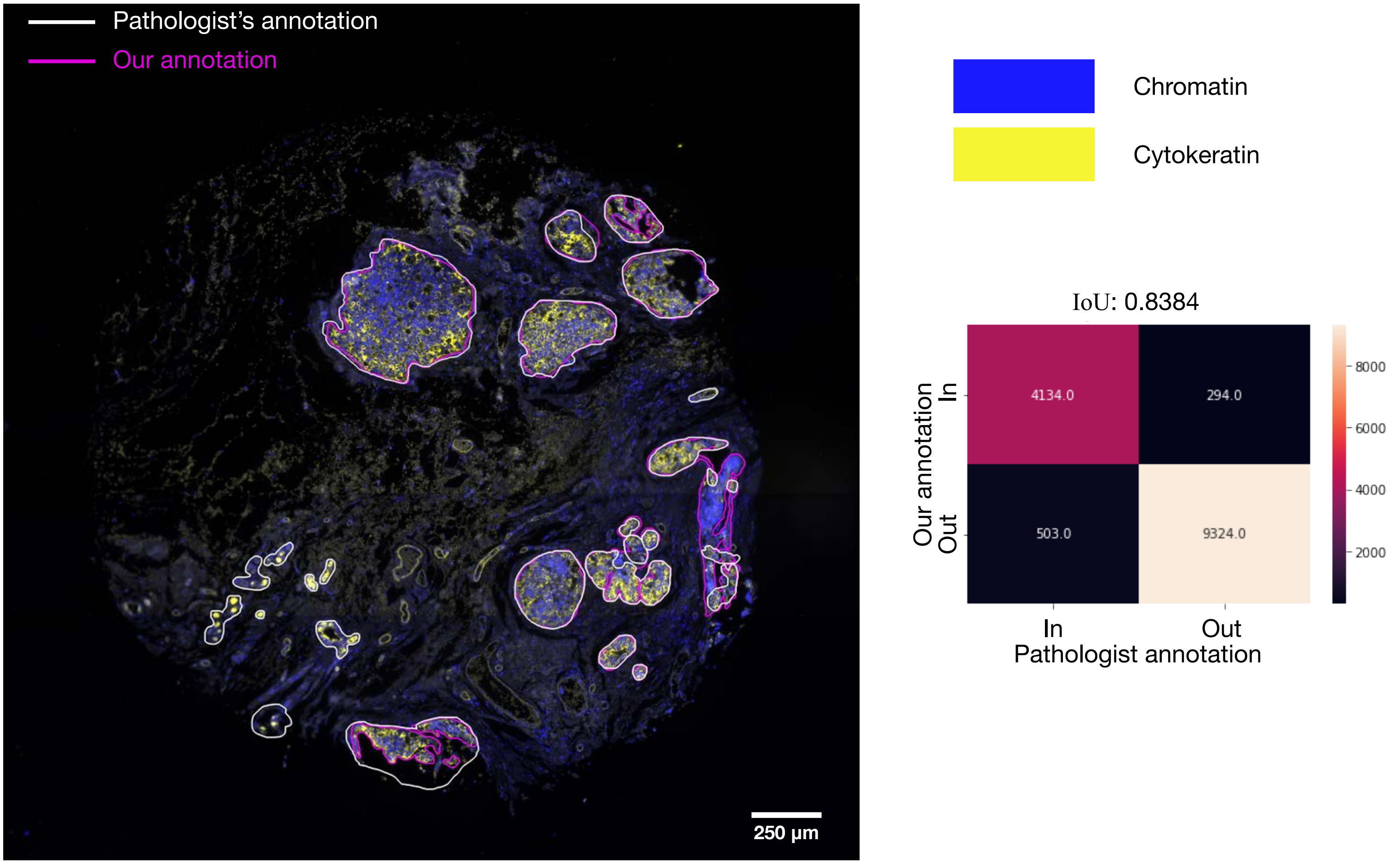

Supplementary Figure 8

Supplementary Figure 8. Comparison of our duct annotation to a pathologist's annotation in DCIS. Our annotation of ducts is outlined in pink and the pathologist's annotation is outlined in white. Heatmaps show the number of cells that are inside or outside of the ducts in our annotation compared to the number of cells in the pathologist's annotation. IoU is the intersection over union that computes the fraction of cells that are assigned with the same annotation by the two annotation sources compared to the total number of cells.

Hyperplasia

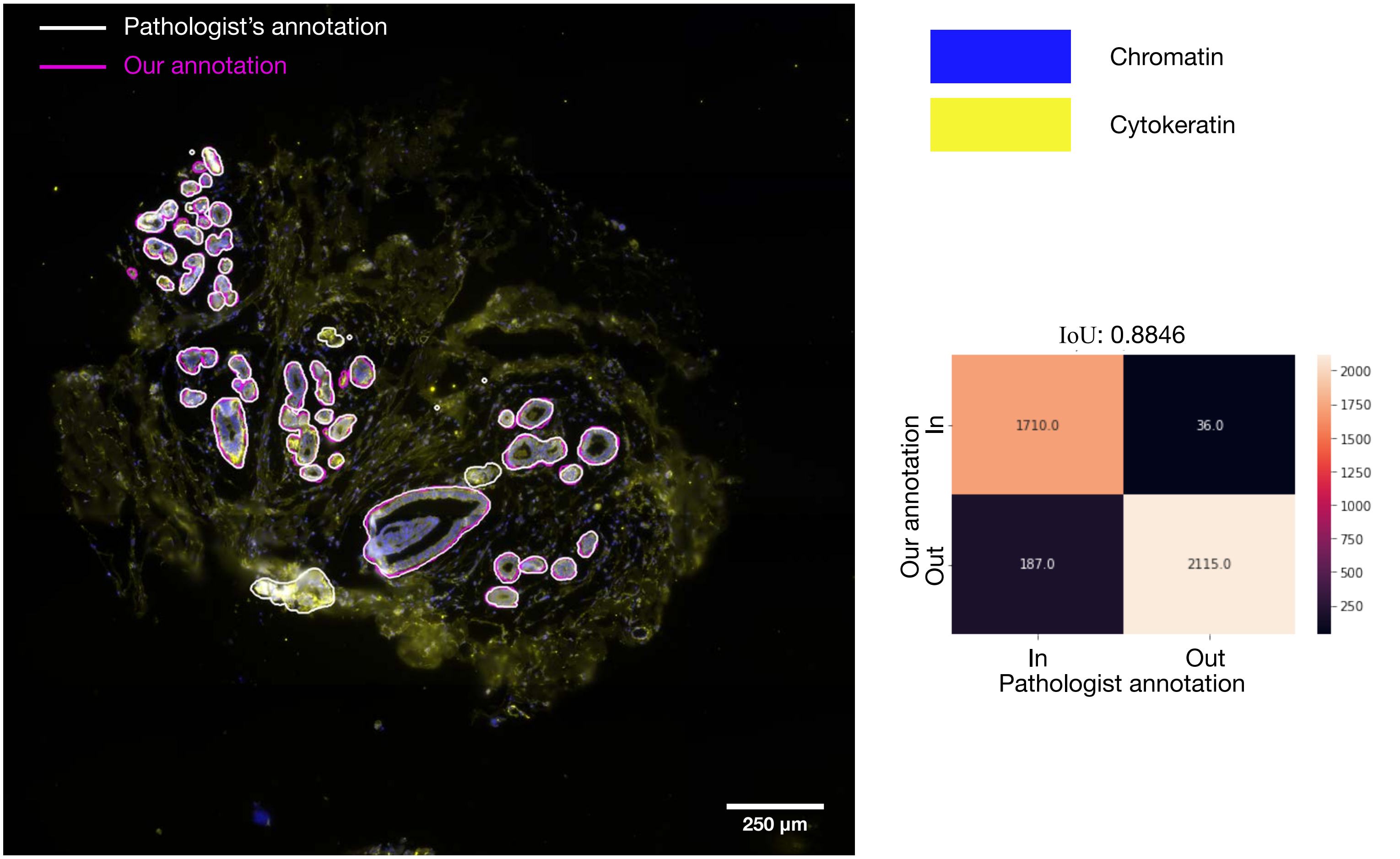

Normal breast tissue

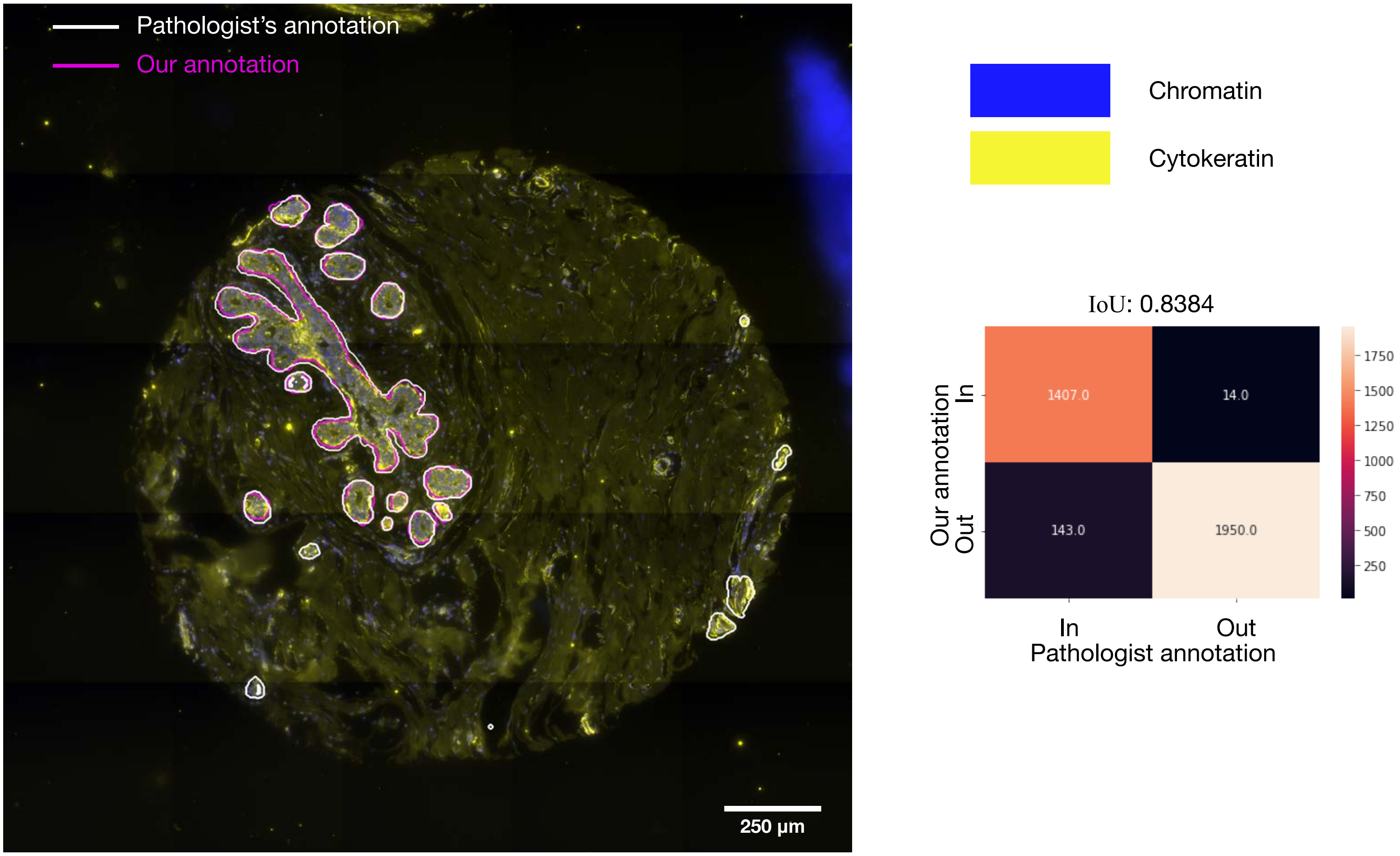

Supplementary Figure 9

Supplementary Figure 9. Comparison of our duct annotation to a pathologist's annotation in Hyperplesia and normal breast tissue. Our annotation of ducts is outlined in pink and the pathologist's annotation is outlined in white. Heatmaps show the number of cells that are inside or outside of the ducts in our annotation compared to the number of cells in the pathologist's annotation. IoU is the intersection over union that computes the fraction of cells that are assigned with the same annotation by the two annotation sources compared to the total number of cells.

Training and validation losses

a Convolutional VAE

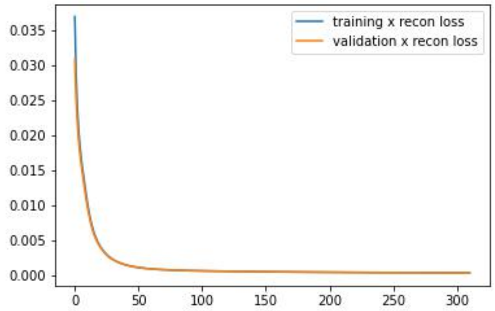

b Convolutional VAE inputs and reconstructions

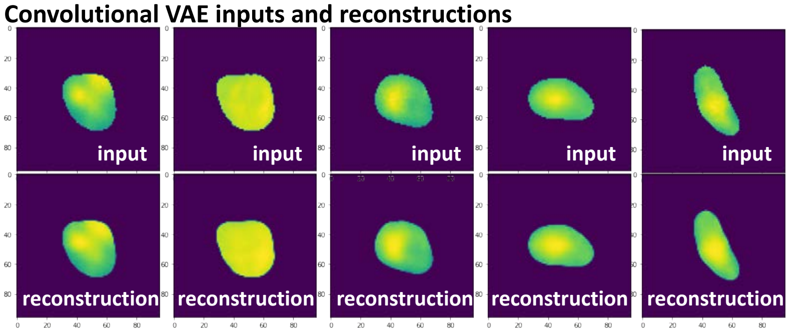

Supplementary Figure 10

Supplementary Figure 10. Convolutional VAE training.
(a) Training and validation losses of the convolutional VAE model over the training epochs (Methods).
(b) Randomly selected examples of the held-out nuclear images and the corresponding reconstruction by the convolutional VAE model.

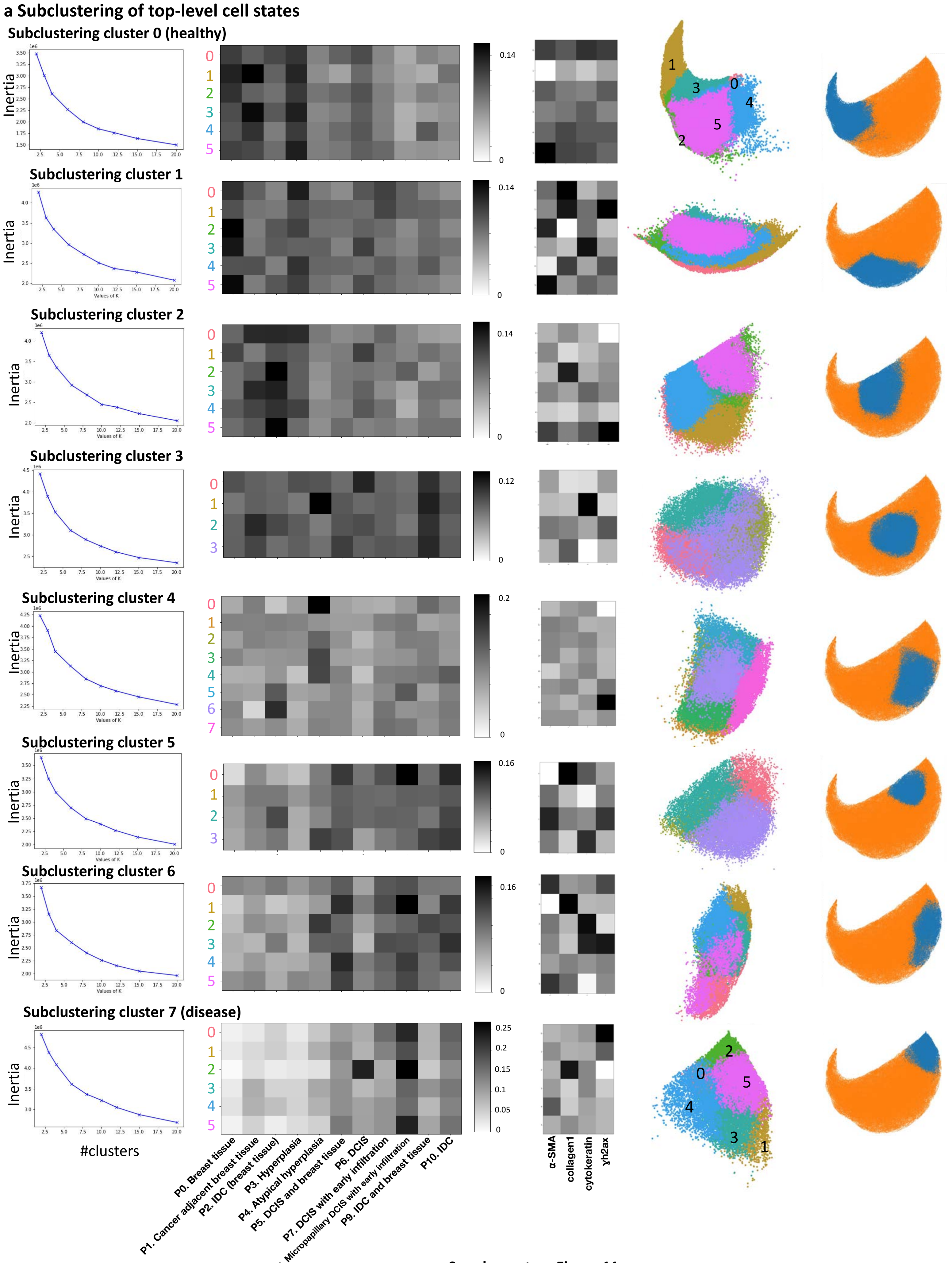

Supplementary Figure 11

b Examples of nuclei in the subclusters of top-level cell states

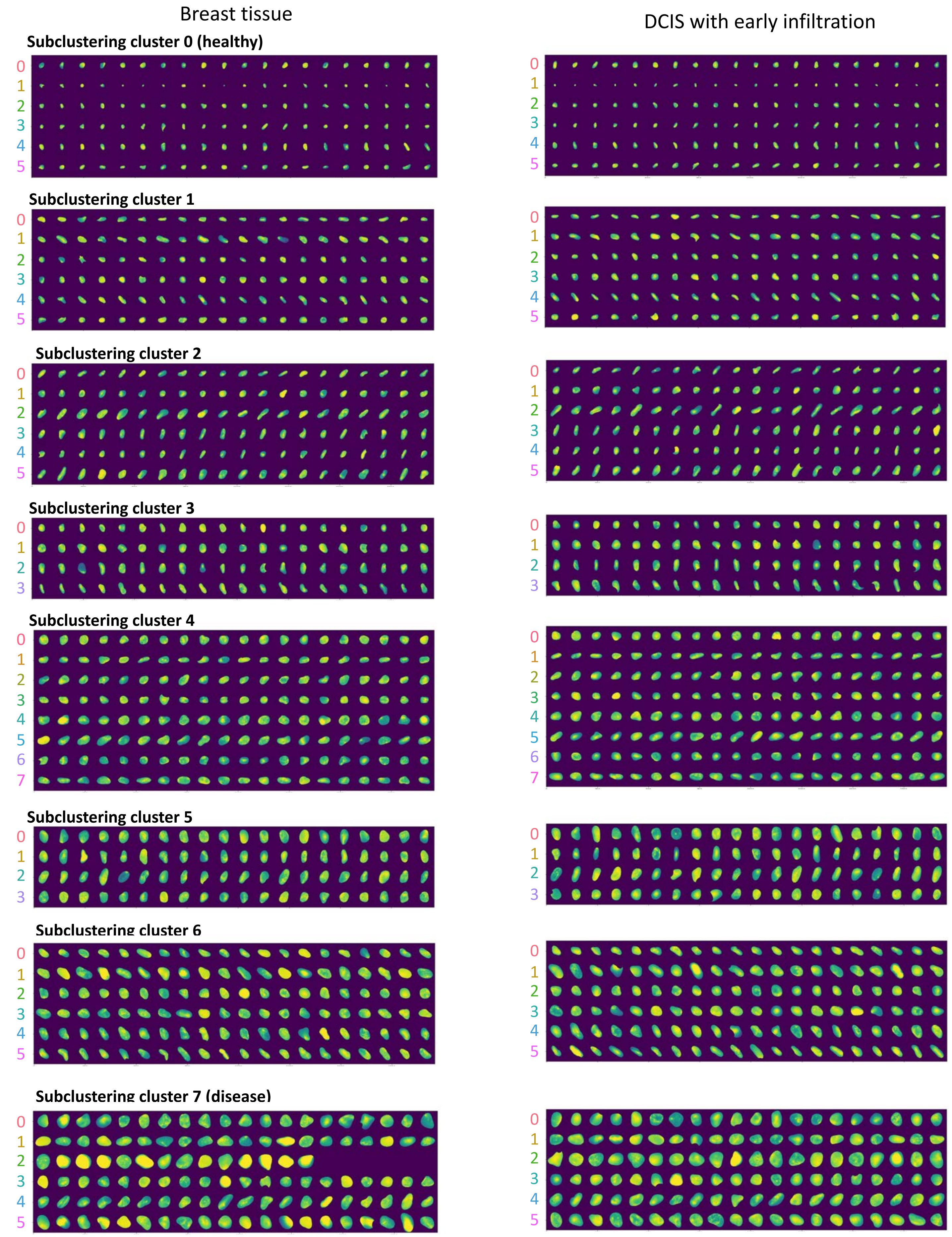

Supplementary Figure 11 (continued)

Supplementary Figure 11. Subclusters of the eight top-level clusters.

(a) Inertia curve as a function of the number of subclusters is shown for each top-level cluster, where inertia is defined as the sum of squared distances of the cells in a particular cluster to the center of that cluster. The other plots from left to right are the proportion of the phenotypic categories in each subcluster, the average protein expression in each subcluster, a UMAP of the subclusters, and the location of the cells in that cluster (blue dots) relative to all cells not in that cluster (orange dots). The UMAP coordinates of each cell are the same as in Figure 2a.

(b) Randomly selected examples of nuclei in each of the eight clusters in two representative phenotypic categories.

a Samples used in training the convolutional VAE and k-means clustering

'br1003a_1_cytokeratin_555_aSMA_647_hoechst' all samples, 'br1003a_3_collagen1_647_hoechst' all samples, 'br1003a_4_cytokeratin_555_gh2ax_647_hoechst' selected samples: A1-11; C1-11; I1-11 'br301_4_cytokeratin_555_aSMA_647_hoechst' all samples, 'br301_6_collagen1_647_hoechst' selected samples: A1-7, B1-7, C1-7, D1-7, E1-7, 'br8018a_1_cytokeratin_555_aSMA_647_hoechst' all samples, 'br8018a_3_collagen1_647_hoechst' all samples, 'br8018a_4_cytokeratin_555_gh2ax_647_hoechst': selected samples: A1-11, B1-11, F1-11; H1-9

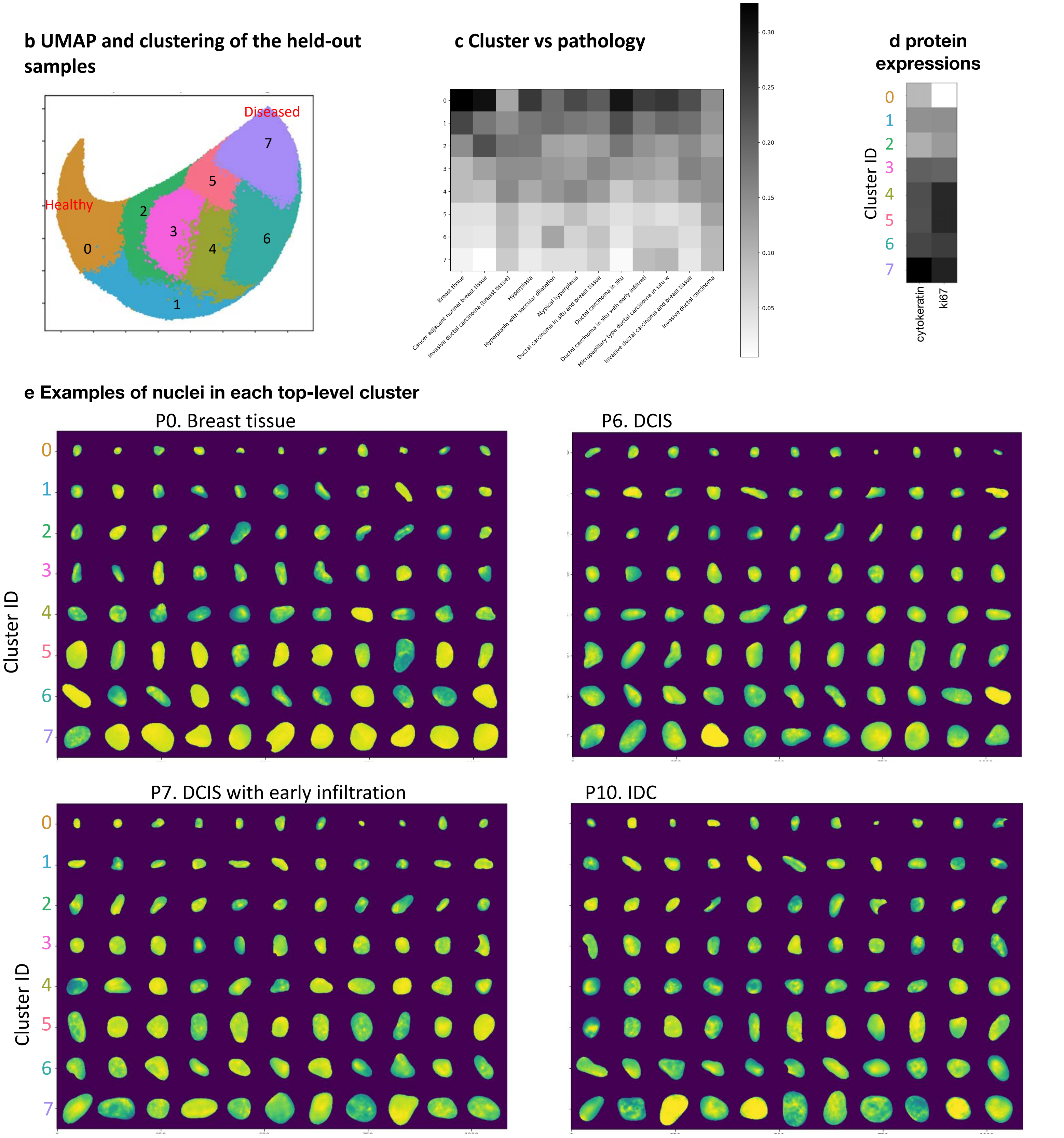

Supplementary Figure 12

Supplementary Figure 12. Results on held-out samples.
(a) Samples used in training the convolutional VAE and k-means clustering.
(b) UMAP of the held-out samples, colored by k-means clustering results, using the same k-means estimator computed with the training samples.
(c) The fraction of cells in each of the eight top-level clusters in each phenotypic category. Columns are normalized to sum to 1.
(d) The expression of each protein marker in each of the eight clusters. Columns are normalized to sum to 1.
(e) Randomly selected examples of nuclei in each of the eight clusters in four representative phenotypic categories.

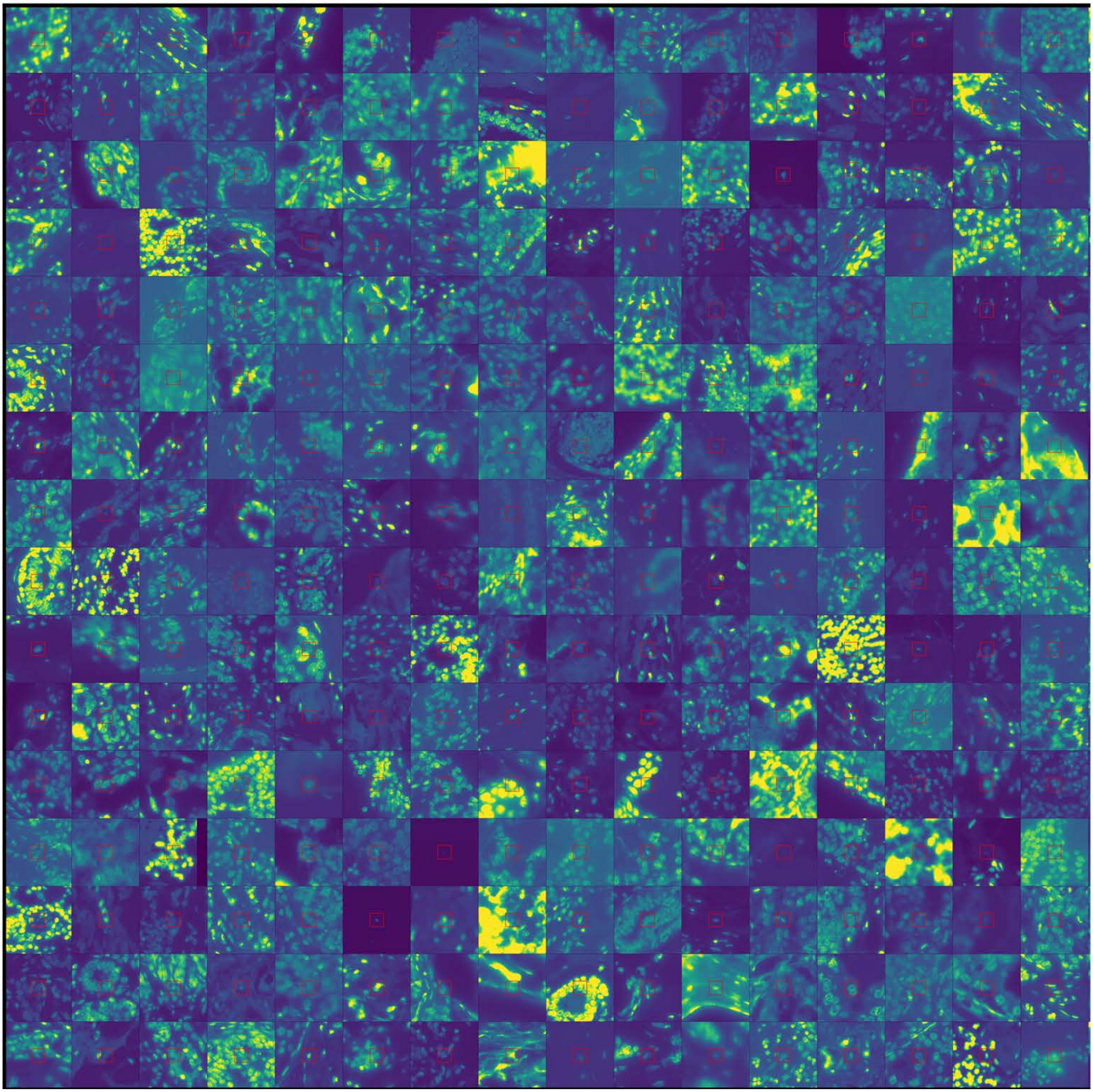

Supplementary Figure 13

Supplementary Figure 13. 256 randomly selected nuclei and their surrounding tissue patches. The queried nucleus is indicated by a red box at the center of each patch.

a Numbers of nuclei assigned with each grade in our clusters

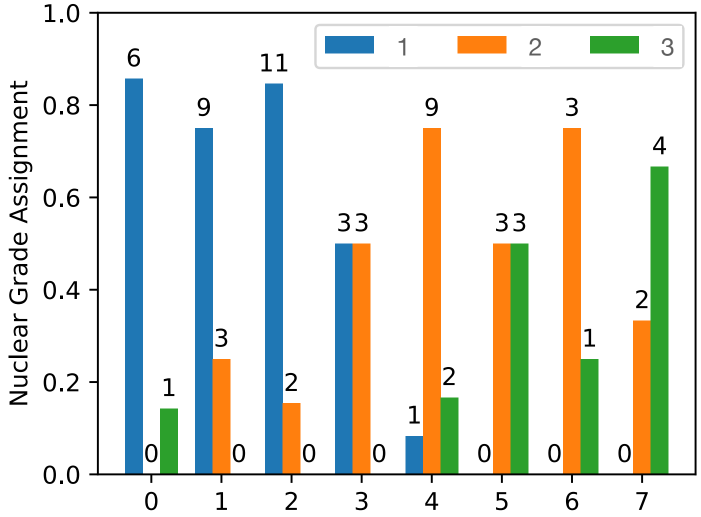

b Number of nuclei assigned with each grade in the three disease stages

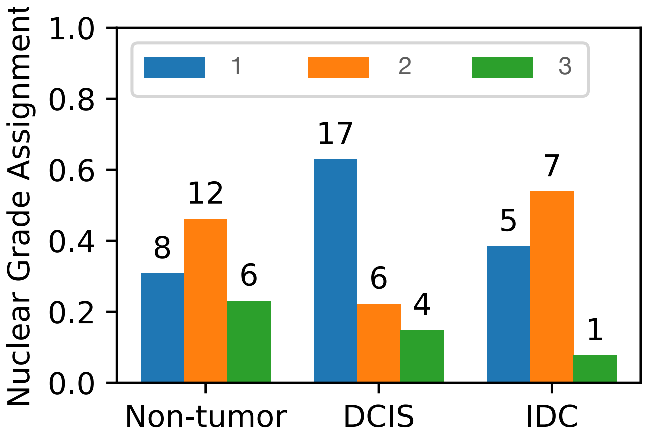

c Nuclear grades assigned by pathologist

NA	2	1	NA	1	1	NA	1	3	3	3	1	2	NA	NA	1
1	1	2	2	1	3	NA	1	NO	NO	NO	1	1	1	NO	1
2	1	2	2	1	NA	2	1	2	1	3	NA	NA	NA	3	NO
1	NO	2	1	NO	1	2	3	1	1	2	2	1	NA	NA	2
2	2	1	3	2	NA	2	2	3	1	NO	3	3	2	1	1
1	3	3	2	2	NA	NO	2	2	2	3	3	1	NO	NO	1
1	1	1	NA	2	3	3	2	2	1	1	NA	NA	NA	1	2
3	1	1	2	3	1	NA	NA	2	2	2	NA	1	1	NO	2
NA	1	NA	1	1	NO	3	1	2	NA	NO	1	2	NO	2	1
2	1	2	3	3	1	2	1	NO	NA	3	NA	2	1	2	2
1	2	1	1	NO	NO	2	1	1	1	2	NA	1	2	2	2
2	3	3	1	NA	2	2	3	2	NA	NA	3	NA	NA	NA	2
2	3	1	2	NA	2	NO	NA	3	1	2	NO	2	NA	NO	2
1	2	3	2	3	NA	NA	NA	2	2	1	2	3	2	NA	3
2	2	1	NA	2	NA	2	NA	2	1	1	3	3	2	NA	NA
1	NO	2	2	1	2	3	NA	1	2	NA	2	1	3	1	NA

d Cluster ID assigned by our model

1	7	3	2	3	1	4	0	0	3	6	0	5	6	0	2
1	1	3	7	7	1	2	3	1	4	1	7	2	2	3	3
4	1	4	2	2	2	3	1	3	6	5	0	0	1	4	0
6	0	1	3	2	2	7	6	1	4	4	7	6	6	0	0
5	2	5	6	0	0	3	4	4	3	1	7	0	1	1	5
4	2	0	0	2	1	4	4	7	0	6	7	1	0	5	5
3	1	2	2	0	3	3	1	6	7	5	5	4	2	3	3
3	0	5	0	3	5	7	4	1	6	7	0	2	3	4	0
4	3	6	5	3	6	3	7	5	5	3	0	2	4	0	5
7	6	6	1	2	4	3	3	2	3	5	4	7	5	2	4
2	0	6	4	2	5	2	6	2	7	3	2	2	1	6	2
3	3	6	5	1	7	5	4	2	5	6	5	5	3	3	7
2	4	0	6	6	0	1	2	1	1	0	3	1	5	5	6
1	3	4	1	1	5	0	2	1	4	0	4	7	2	4	5
7	3	3	0	2	1	6	7	3	2	2	0	1	6	3	1
2	3	6	4	7	2	5	0	2	6	0	4	0	2	7	0

e Disease stages obtained from Biomax

Non-tumor	Non-tumor	DCIS	Non-tumor	Non-tumor	Non-tumor	Non-tumor	DCIS	DCIS	Non-tumor	DCIS	IDC	IDC	IDC	IDC	Non-tumor
DCIS	DCIS	Non-tumor	IDC	Non-tumor	IDC	Non-tumor	DCIS	DCIS	Non-tumor	DCIS	DCIS	IDC	DCIS	Non-tumor	Non-tumor
Non-tumor	Non-tumor	Non-tumor	DCIS	DCIS	DCIS	IDC	DCIS	Non-tumor	DCIS	Non-tumor	Non-tumor	DCIS	Non-tumor	Non-tumor	Non-tumor
DCIS	DCIS	Non-tumor	Non-tumor	Non-tumor	Non-tumor	DCIS	DCIS	Non-tumor	DCIS	DCIS	DCIS	Non-tumor	DCIS	Non-tumor	Non-tumor
DCIS	Non-tumor	DCIS	DCIS	DCIS	Non-tumor	DCIS	DCIS	DCIS	IDC	DCIS	Non-tumor	Non-tumor	DCIS	DCIS	IDC
IDC	Non-tumor	Non-tumor	IDC	DCIS	Non-tumor	IDC	DCIS	Non-tumor	Non-tumor	Non-tumor	Non-tumor	Non-tumor	IDC	IDC	Non-tumor
Non-tumor	Non-tumor	Non-tumor	DCIS	Non-tumor	Non-tumor	DCIS	Non-tumor	DCIS	Non-tumor	DCIS	Non-tumor	Non-tumor	DCIS	IDC	DCIS
DCIS	DCIS	Non-tumor	DCIS	Non-tumor	IDC	DCIS	IDC	DCIS	Non-tumor	Non-tumor	Non-tumor	IDC	Non-tumor	Non-tumor	DCIS
DCIS	DCIS	DCIS	Non-tumor	Non-tumor	Non-tumor	Non-tumor	DCIS	DCIS	Non-tumor	Non-tumor	DCIS	Non-tumor	Non-tumor	Non-tumor	IDC
Non-tumor	IDC	DCIS	IDC	Non-tumor	DCIS	DCIS	IDC	Non-tumor	IDC	DCIS	Non-tumor	IDC	Non-tumor	Non-tumor	Non-tumor
DCIS	DCIS	DCIS	Non-tumor	DCIS	DCIS	DCIS	DCIS	DCIS	DCIS	DCIS	Non-tumor	Non-tumor	Non-tumor	Non-tumor	IDC
DCIS	DCIS	Non-tumor	Non-tumor	DCIS	IDC	Non-tumor	Non-tumor	Non-tumor	Non-tumor	Non-tumor	IDC	Non-tumor	DCIS	Non-tumor	Non-tumor
Non-tumor	Non-tumor	Non-tumor	Non-tumor	IDC	DCIS	IDC	Non-tumor	Non-tumor	DCIS	IDC	DCIS	DCIS	IDC	DCIS	Non-tumor
Non-tumor	DCIS	DCIS	Non-tumor	IDC	Non-tumor	Non-tumor	Non-tumor	IDC	DCIS	IDC	DCIS	DCIS	DCIS	Non-tumor	DCIS
Non-tumor	Non-tumor	Non-tumor	IDC	Non-tumor	IDC	DCIS	Non-tumor	Non-tumor	DCIS	DCIS	IDC	IDC	DCIS	Non-tumor	DCIS
Non-tumor	Non-tumor	DCIS	Non-tumor	Non-tumor	Non-tumor	Non-tumor	DCIS	IDC	DCIS	Non-tumor	Non-tumor	IDC	IDC	DCIS	DCIS

Supplementary Figure 14

Supplementary Figure 14. Severity of pathologist-assigned nuclear grade is positively correlated with cell state malignancy inferred by our model.

(a) The number of nuclei assigned by a pathologist with each of the three grades in the eight top-level clusters identified by our model.

(b) The number of nuclei assigned with each of the three pathologist-assigned grades in the three disease stages.

(c) Pathologist-assigned nuclear grades of the nuclei bounded by the red boxes. Nuclei are graded from 1 to 3, where 3 is the most malignant. "NO" means there is no nucleus at the center of the image. "NA" means a grade cannot be assigned because there are multiple nuclei at the center or the nucleus is out of focus.

(d) Cluster ID of the nucleus at the center bounded by the red box.

(e) Disease stage (as assigned by Biomax) of the tissue section containing the queried nucleus.

Pathology classifier training and validation losses (with VAE latent as the inputs)

Subclusters of cluster 0

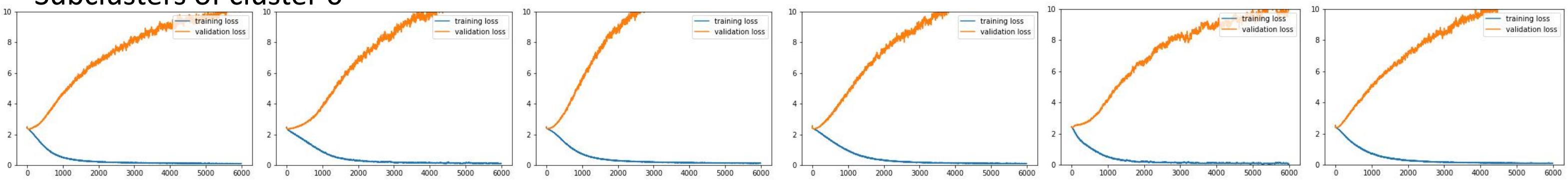

Subclusters of cluster 1

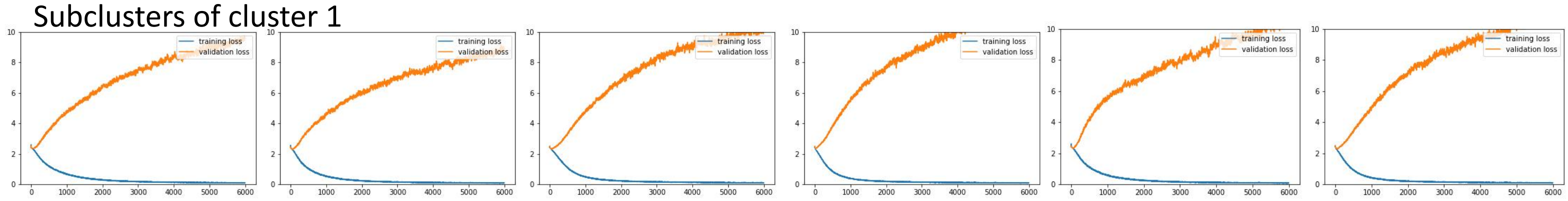

Subclusters of cluster 2

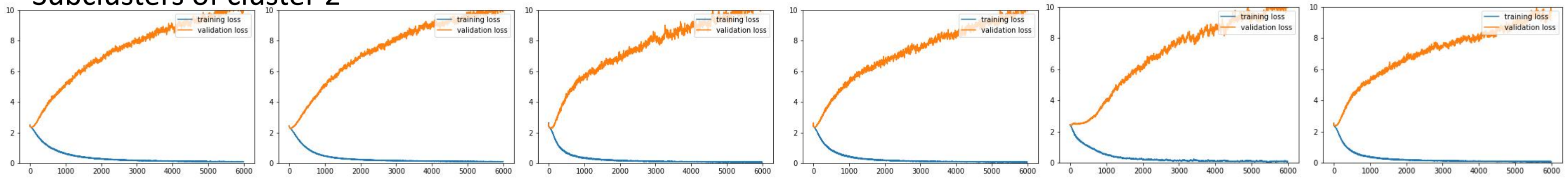

Subclusters of cluster 3

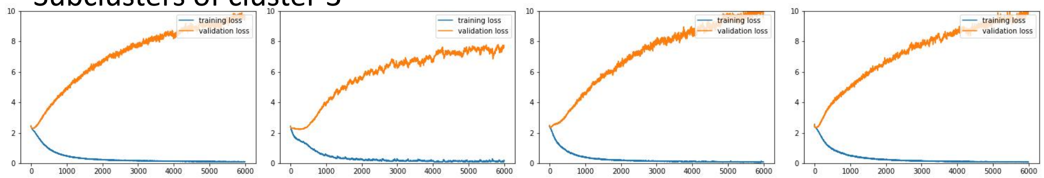

Subclusters of cluster 4

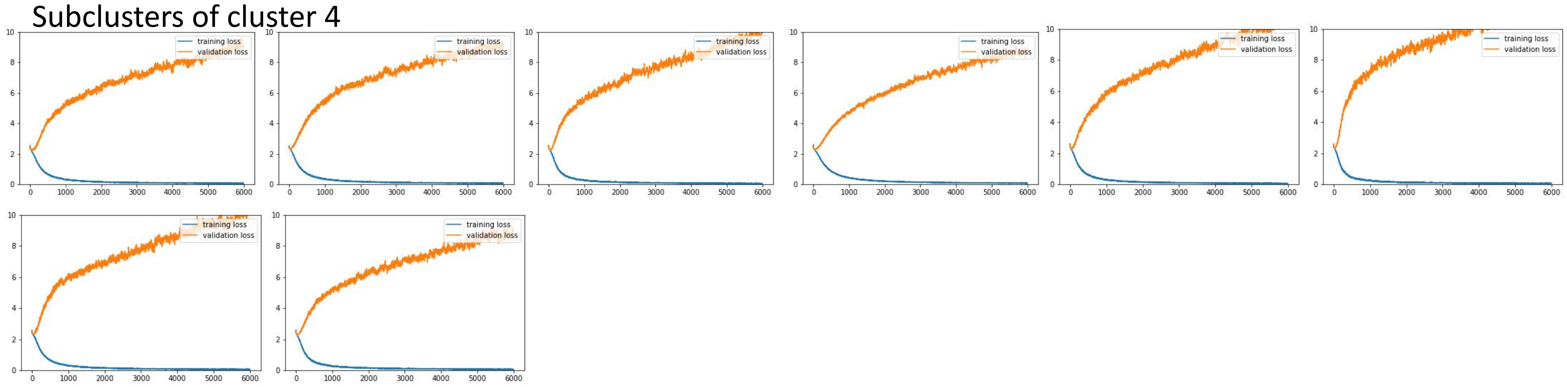

Subclusters of cluster 5

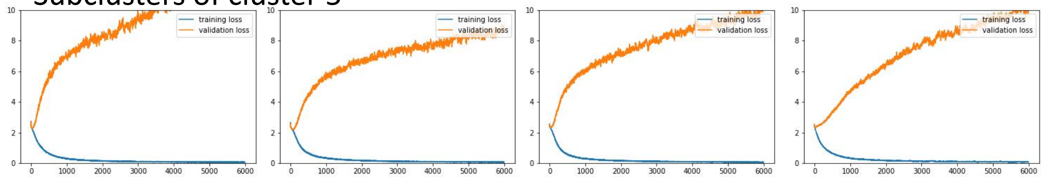

Subclusters of cluster 6

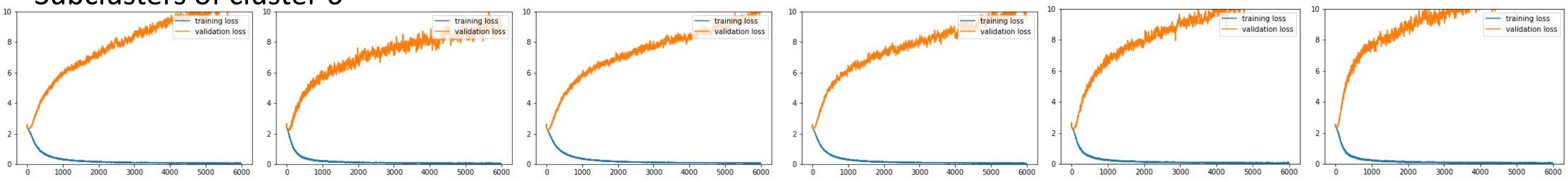

Subclusters of cluster 7

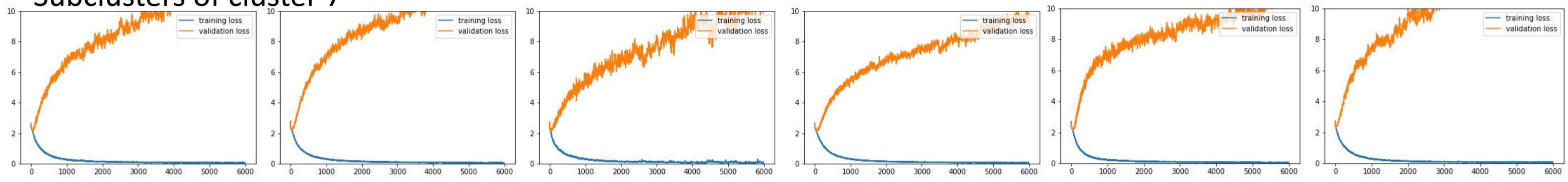

Supplementary Figure 15 a

Pathology classifier confusion matrices (with VAE latent as the input)

Subclusters of cluster 0

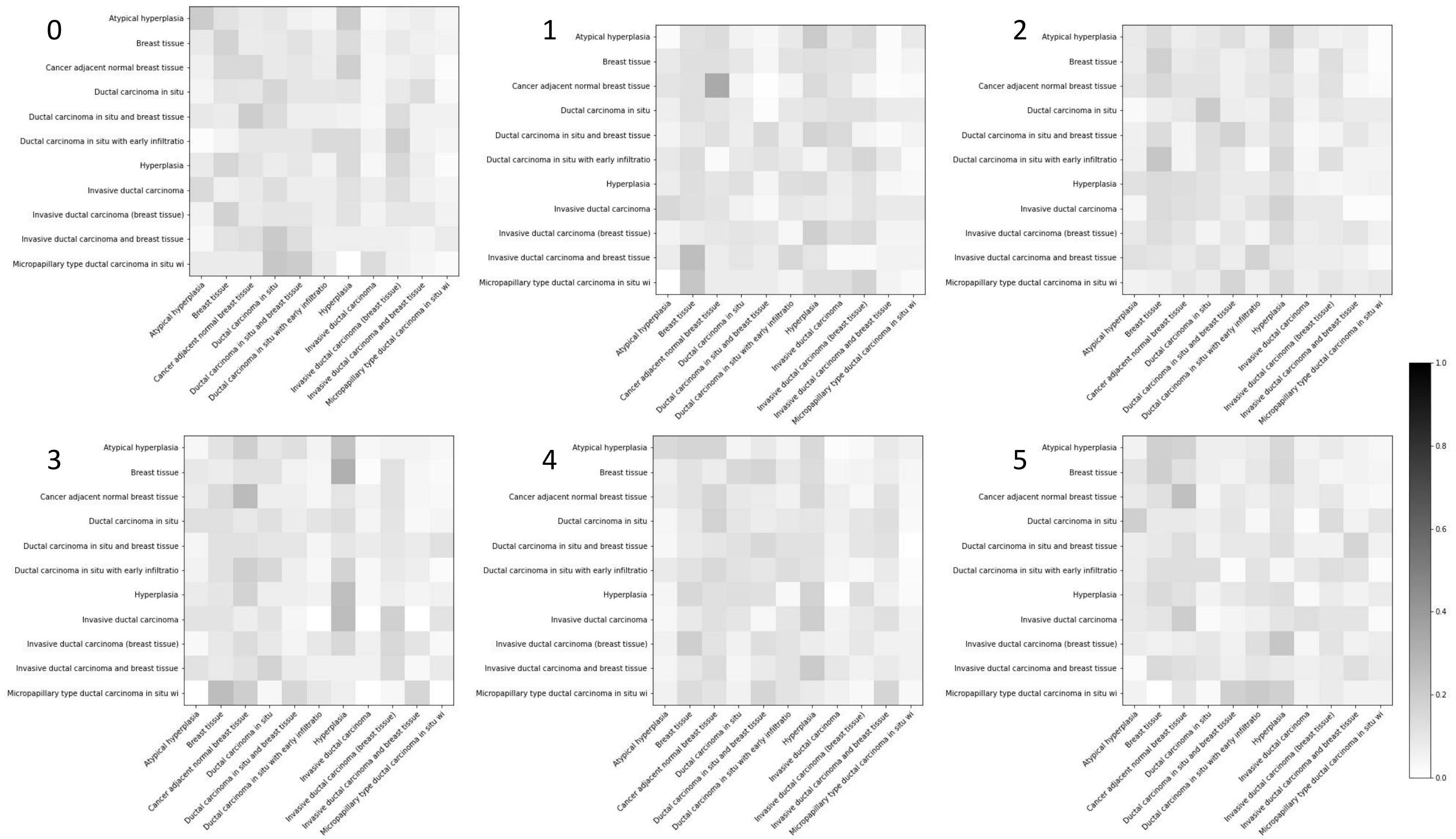

Subclusters of cluster 1

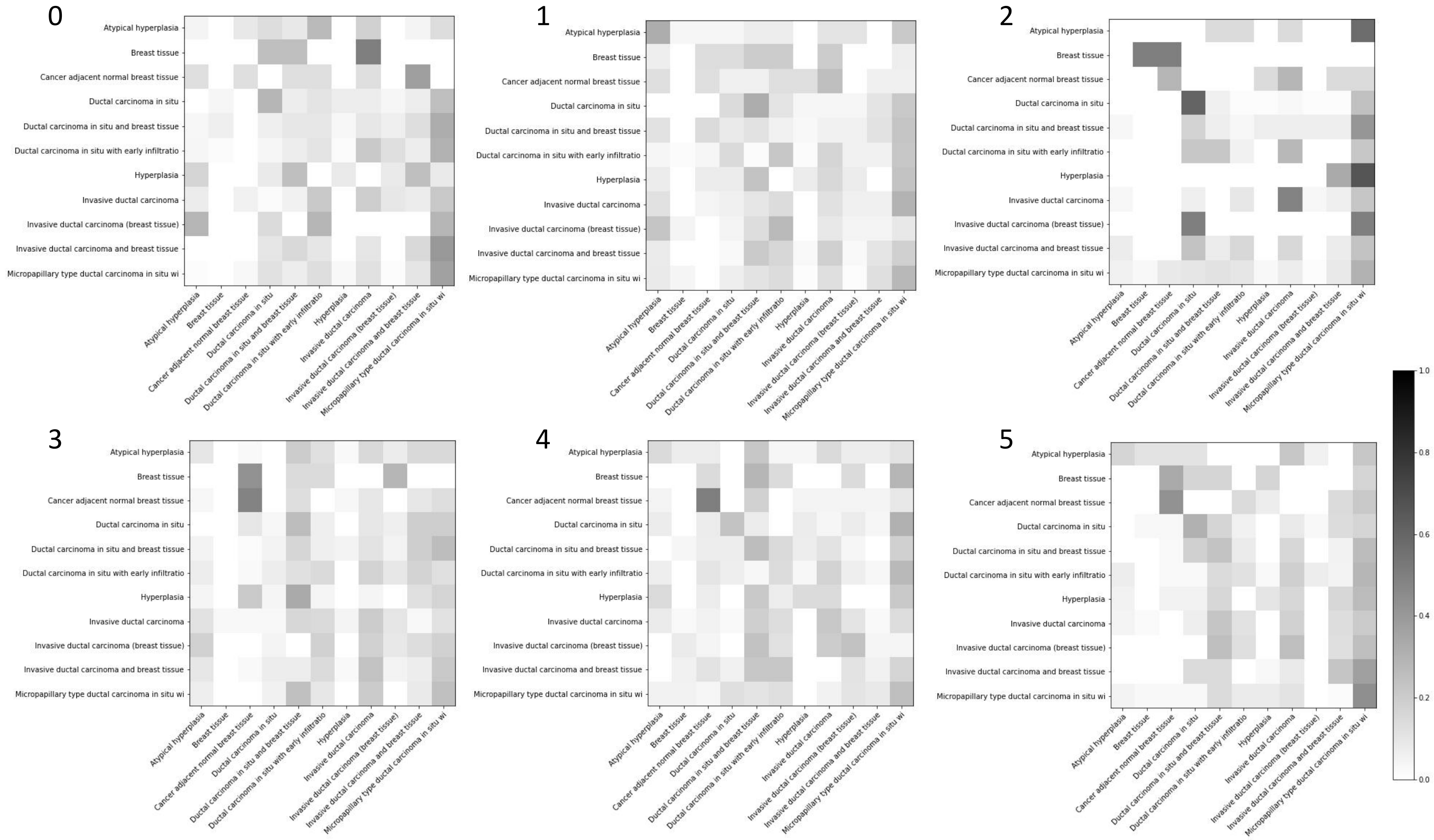

Supplementary Figure 15 b

Pathology classifier confusion matrices (with VAE latent as the input)

Subclusters of cluster 2

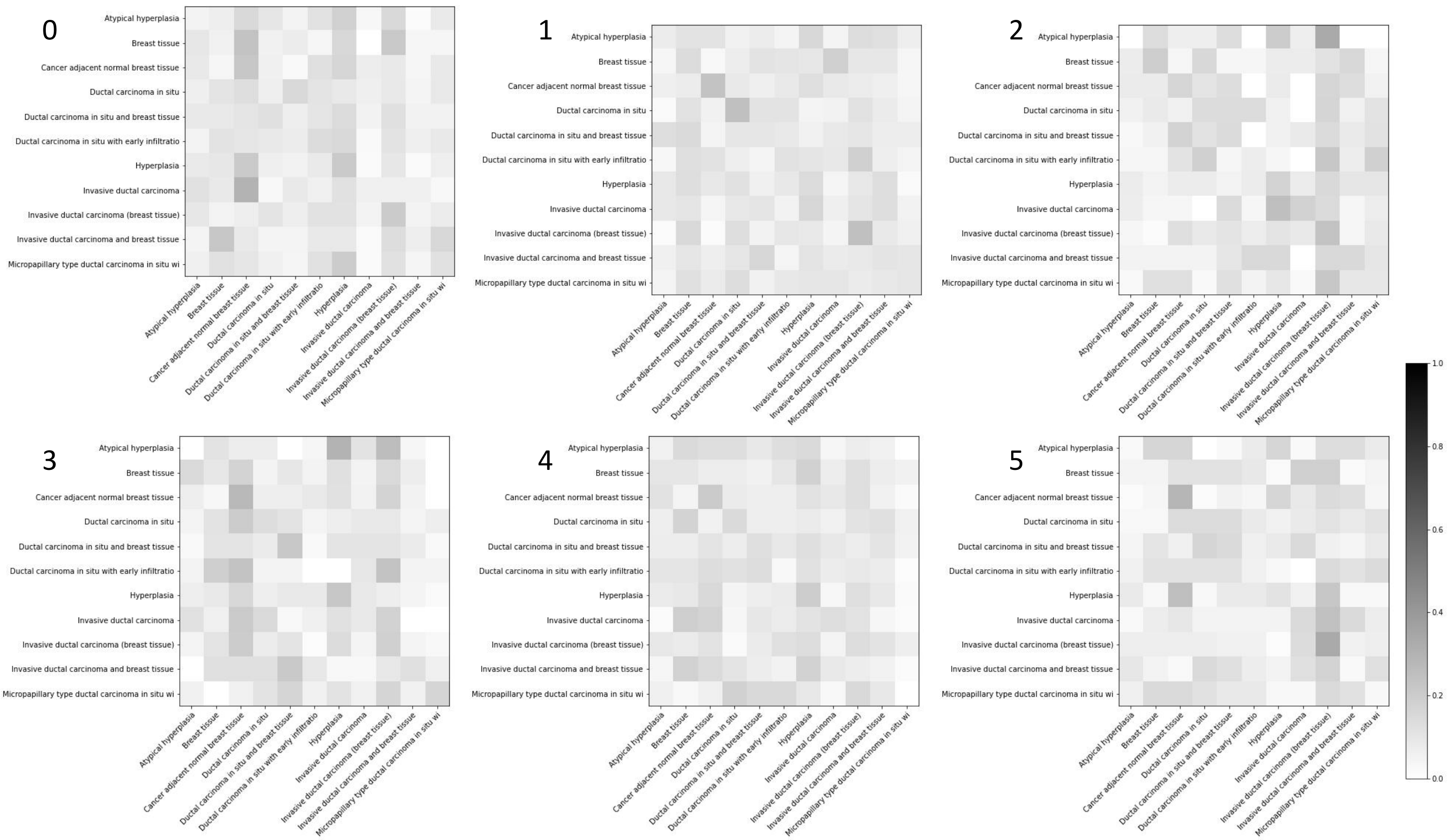

Subclusters of cluster 3

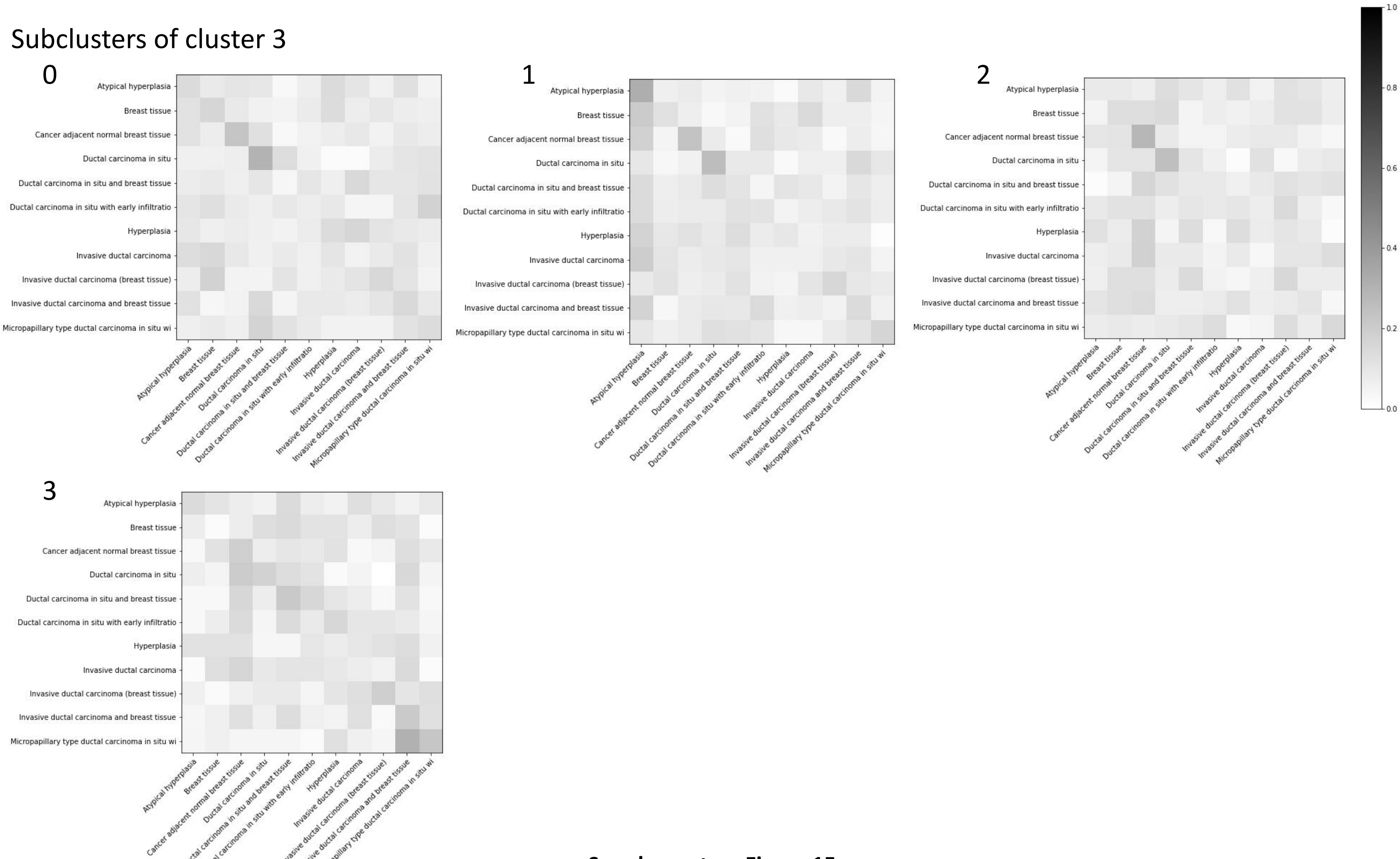

Supplementary Figure 15 c

Pathology classifier confusion matrices (with VAE latent as the input)

Subclusters of cluster 4

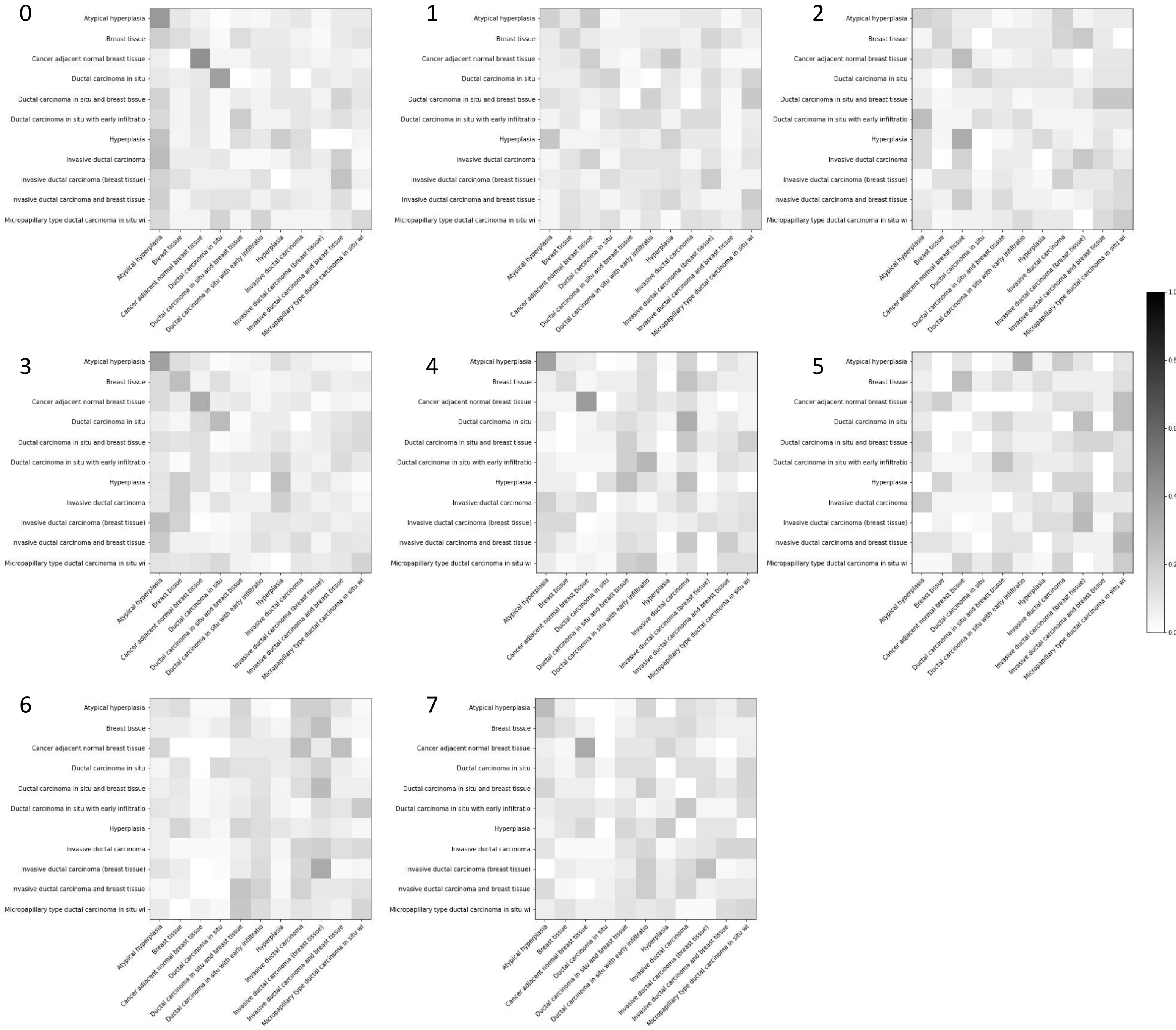

Subclusters of cluster 5

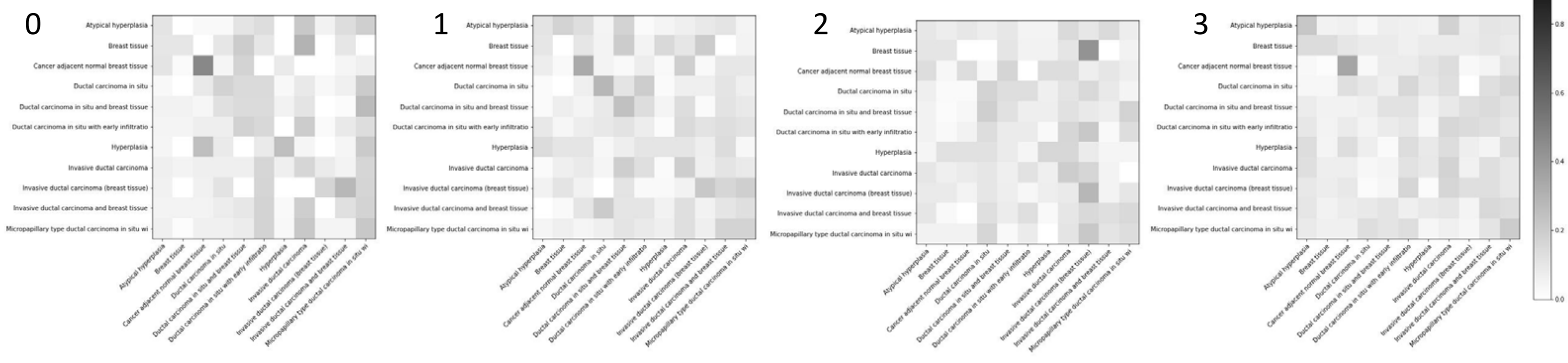

Supplementary Figure 15 d

Pathology classifier confusion matrices (with VAE latent as the input)

Subclusters of cluster 6

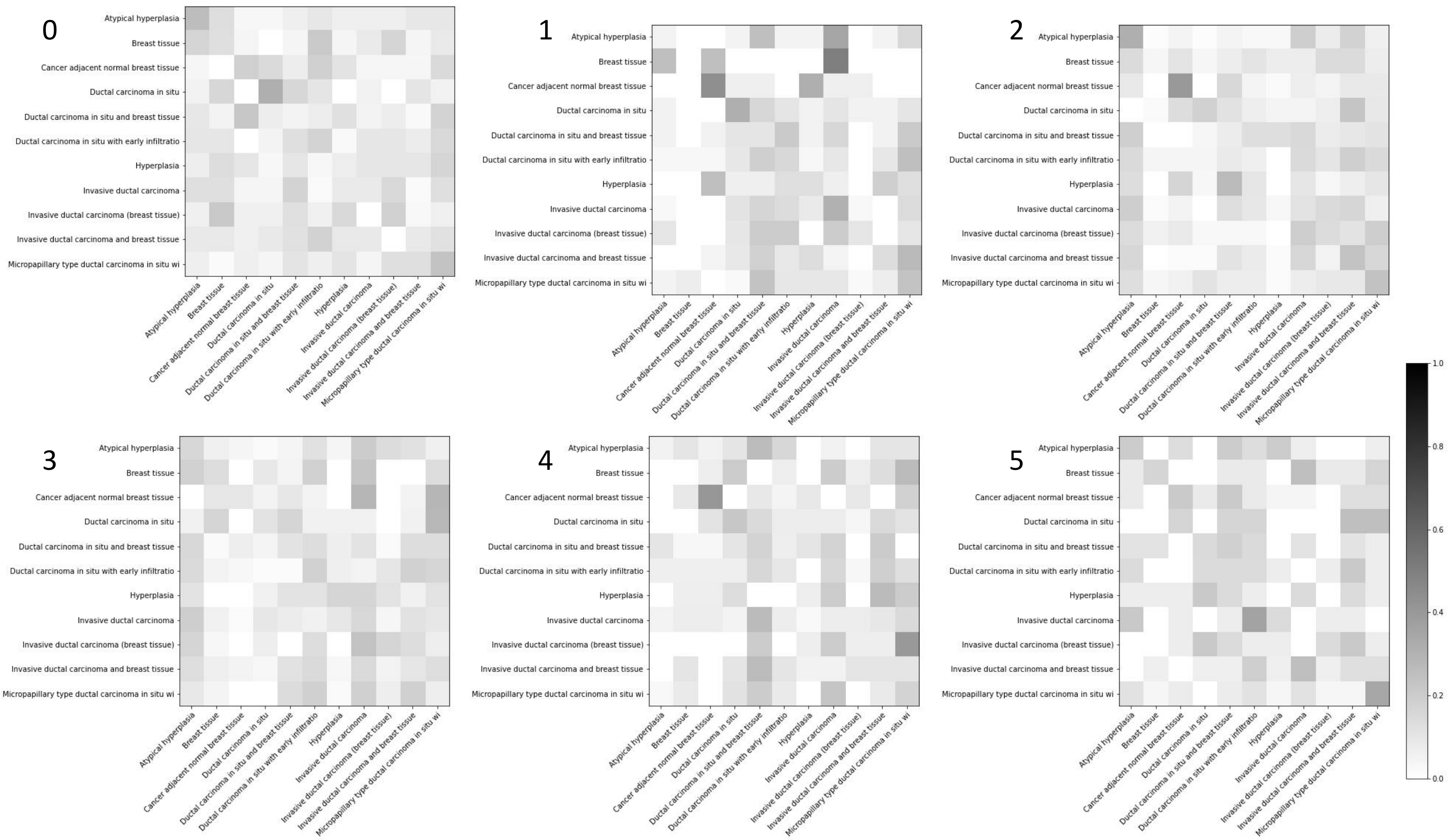

Subclusters of cluster 7

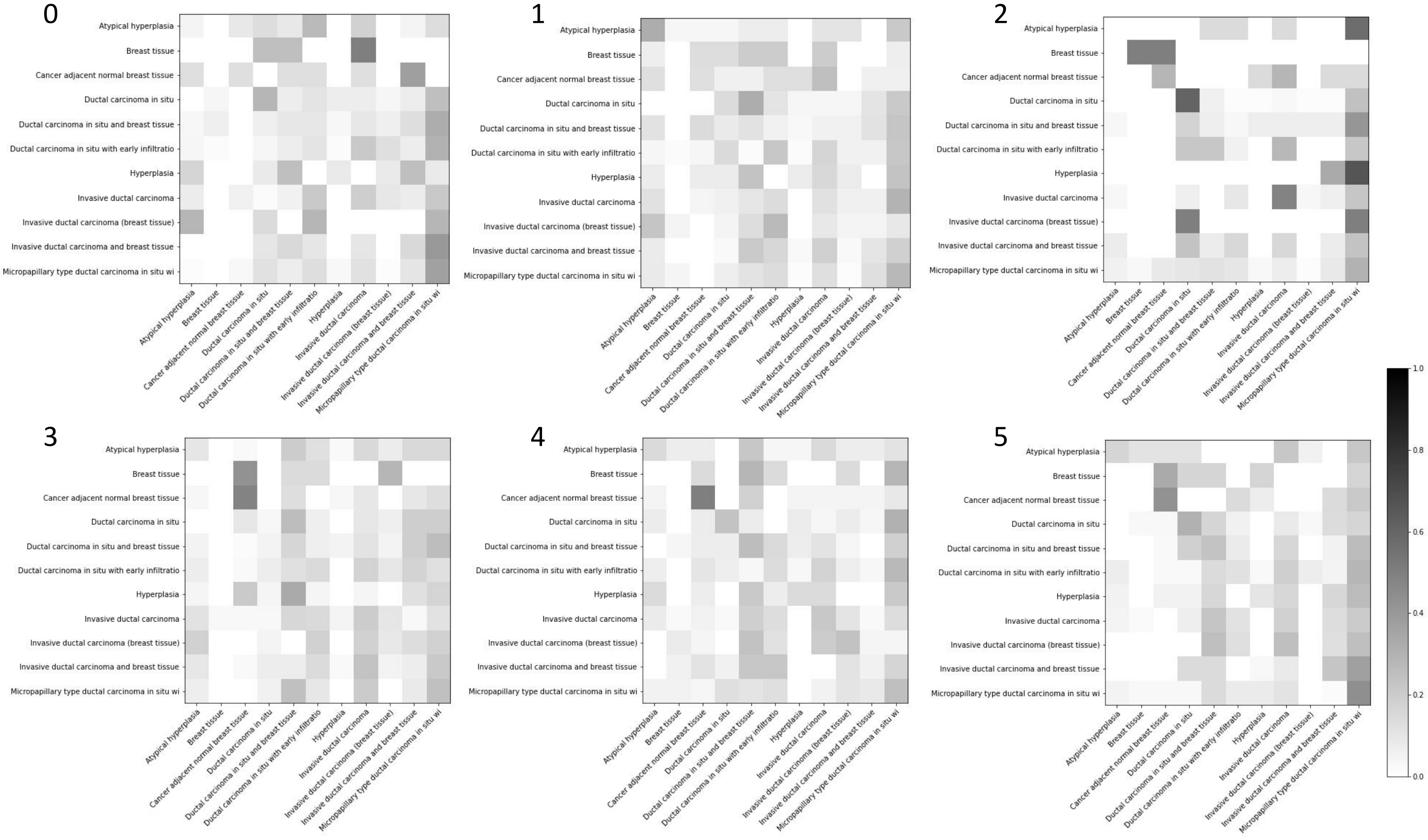

Supplementary Figure 15 e

Supplementary Figure 15. Training curves and confusion matrices of the pathology classifiers that predict the phenotypic category of a cell from its VAE latent space embedding within each subcluster.
(a) Training and validation losses.
(b)-(e) Confusion matrices.

Confusion matrices of classifying cell cluster assignment using NMCO scores

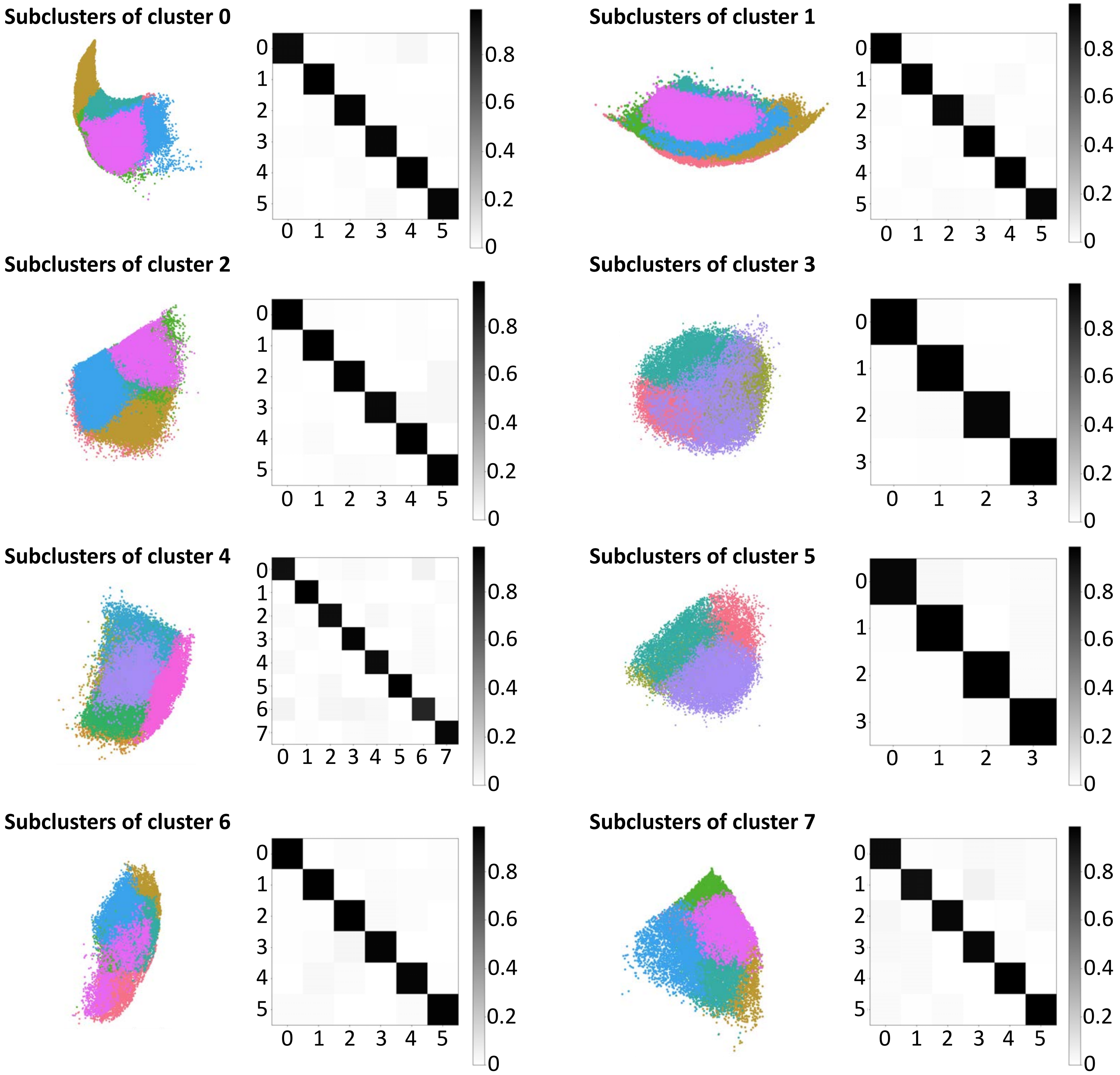

Supplementary Figure 16

Supplementary Figure 16. Confusion matrices for cell cluster assignment using the NMCO features. The subcluster assignment of a cell can be predicted with high accuracy from the NMCO scores of the NMCO features of a cell.

a Minimum correlation = 0.7
Examples of NMCO features in each group

summary	examples
size, curvature	radius, area, length of positive curvature,moments
aspect ratios	ratio of minor and major axes
curvature	average curvature, fraction of positive curvature
dissimilarity	dissimilarity with different offsets
homogeneity	homogeneity, angular second momentum
moments	normalized central image moments
moments	central image moments
moments	central image moments
not grouped	eccentricity, orientation, concavity

b Minimum correlation = 0.75
Examples of NMCO features in each group

summary	examples
size, curvature	radius, area, length of positive curvature
aspect ratios	ratio of minor and major axes
homogeneity	homogeneity, angular second momentum, std of centroid to boundary distance
curvature	average curvature, fraction of positive curvature
dissimilarity	dissimilarity with different offsets
homogeneity	homogeneity, angular second momentum
moments	normalized central image moments
moments	central image moments
moments	central image moments
not grouped	eccentricity, orientation, concavity

c Minimum correlation = 0.8
Examples of NMCO features in each group

summary	examples
size, curvature	radius, area, length of positive curvature
aspect ratios	ratio of minor and major axes
homogeneity	homogeneity, angular second momentum, std of centroid to boundary distance
curvature	average curvature, fraction of positive curvature
dissimilarity	dissimilarity with different offsets
homogeneity	homogeneity, angular second momentum
moments	normalized central image moments
moments	central image moments
moments	central image moments
not grouped	eccentricity, orientation, concavity

d Minimum correlation = 0.85
Examples of NMCO features in each group

summary	examples
size, curvature	radius, area, length of positive curvature
aspect ratios	ratio of minor and major axes
moments	moments_hu, inverse of circularity
curvature	average curvature, fraction of positive curvature
dissimilarity	dissimilarity with different offsets
homogeneity	homogeneity with different offsets
homogeneity	homogeneity, angular second momentum
energy	angular second momentum, energy
correlation	correlation with different offsets
moments	normalized central image moments
moments	normalized weighted central image moments
moments	central image moments
not grouped	eccentricity, orientation, concavity

e Minimum correlation = 0.7

NMCO features with significantly different values in at least one cluster

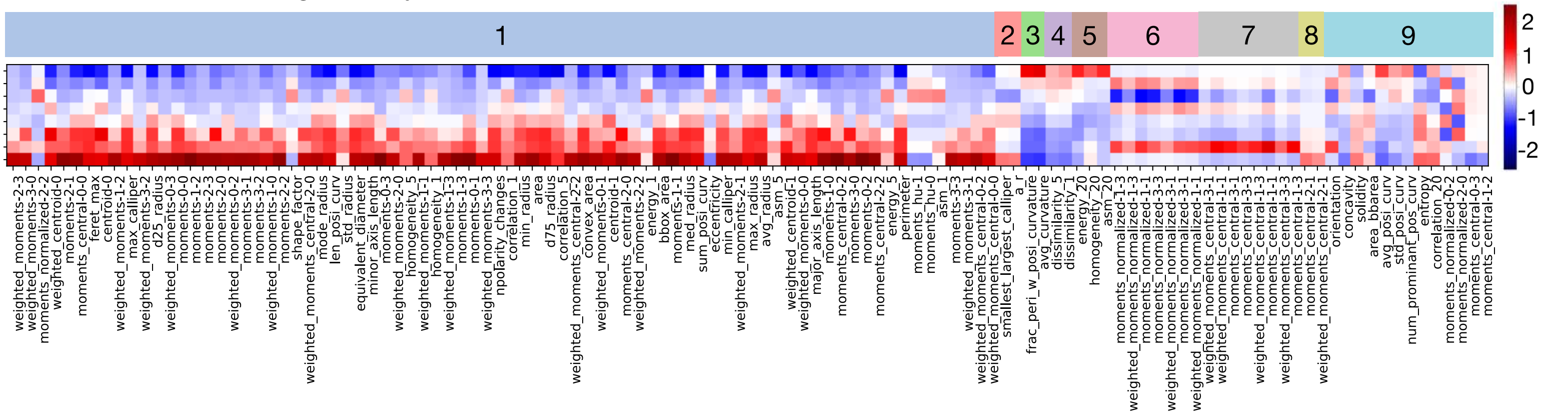

f Minimum correlation = 0.85

NMCO features with significantly different values in at least one cluster

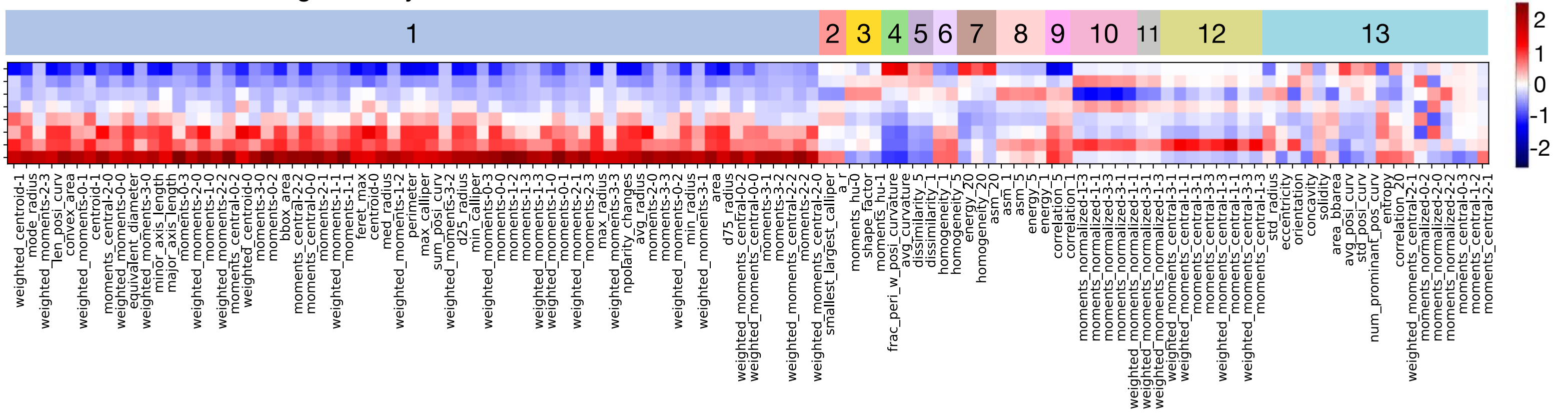

Supplementary Figure 17

Supplementary Figure 17. Grouping of NMCO features at different correlation thresholds. NMCO features that are significantly different in at least one of the eight top-level clusters are grouped by correlation: Each of the 201 NMCO features was tested for whether its mean in any of the eight clusters was different to the mean in cells outside of that cluster (Methods); highly correlated features were grouped together with different thresholds of minimum correlation (Methods).
(a)-(d) Representative examples of NMCO features in each group when different correlation thresholds are used.
(e) The heatmap shows the mean of the significant NMCO features (columns) in each of the eight top-level clusters (rows) ordered by correlation groups. The grouping is shown for a correlation threshold of 0.7.
(f) The heatmap shows the mean of the significant NMCO features (columns) in each of the eight top-level clusters (rows) ordered by correlation groups. The grouping is shown for a correlation threshold of 0.85.

Classifiers trained to distinguish cells inside vs outside of breast ducts from VAE latent

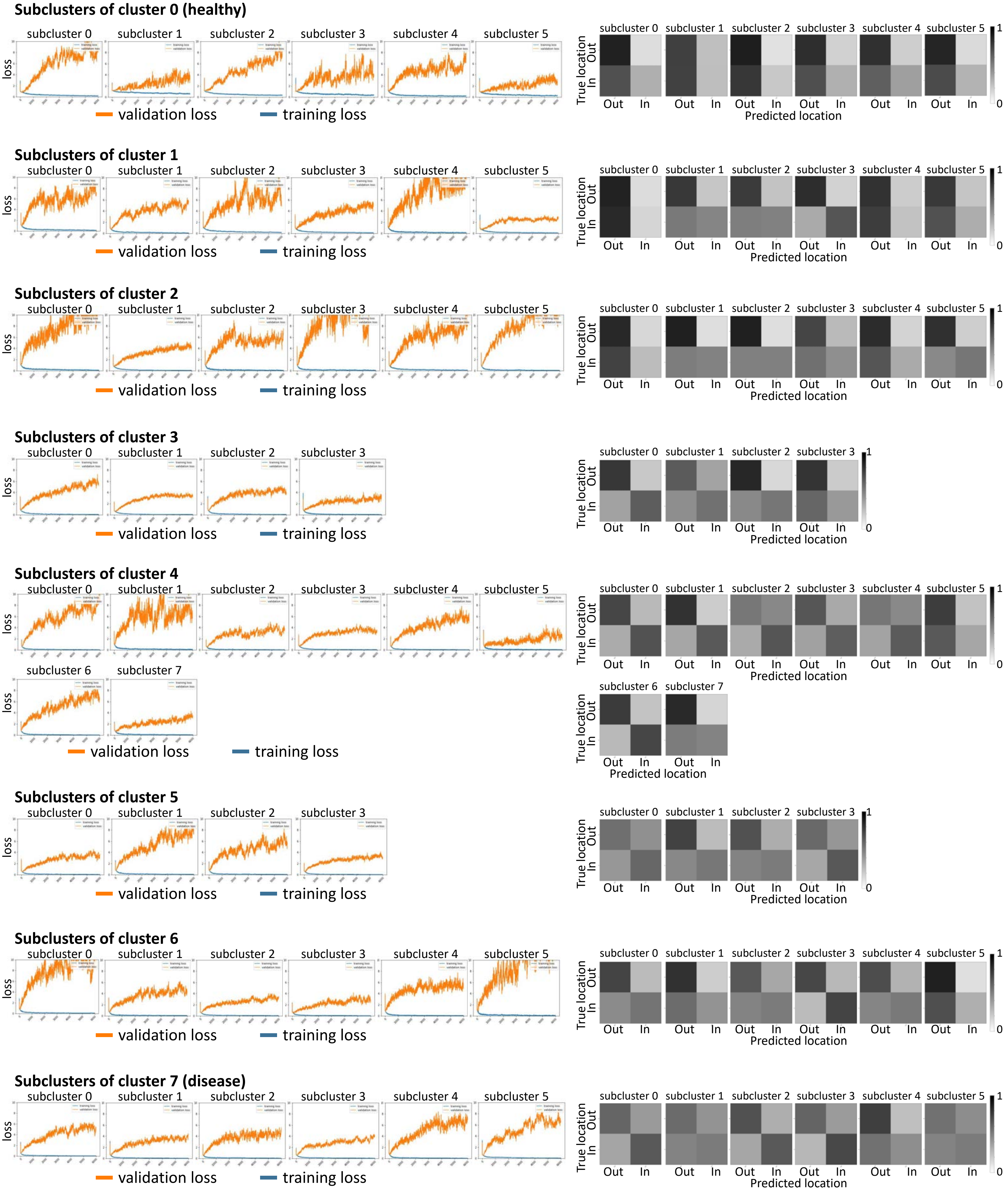

Supplementary Figure 18 a

Classifiers trained to distinguish cells inside vs outside of breast ducts from VAE latent (with manual duct segmentation)

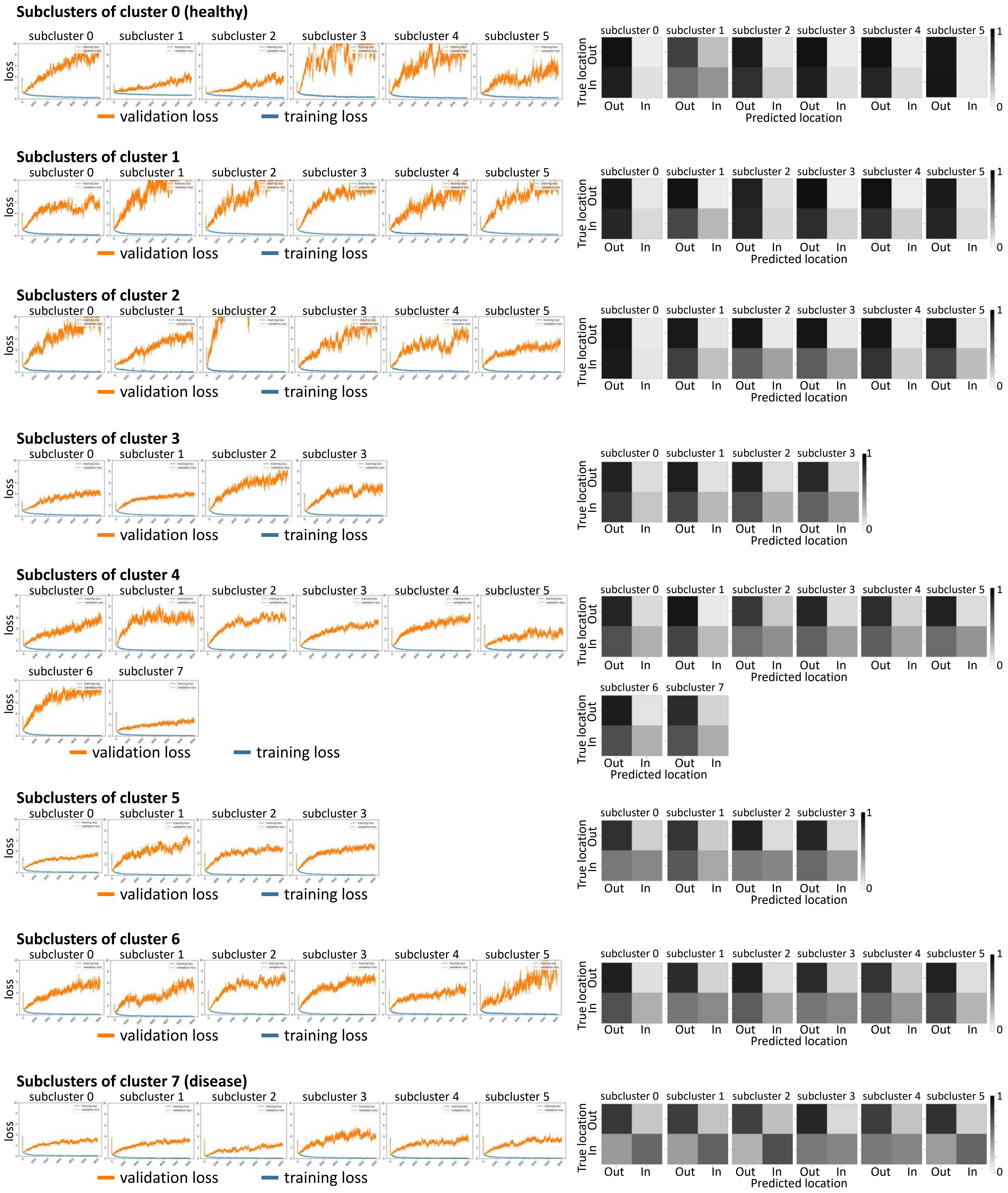

Supplementary Figure 18 b

Classifiers trained to distinguish cells inside vs outside of breast ducts from NMCO features

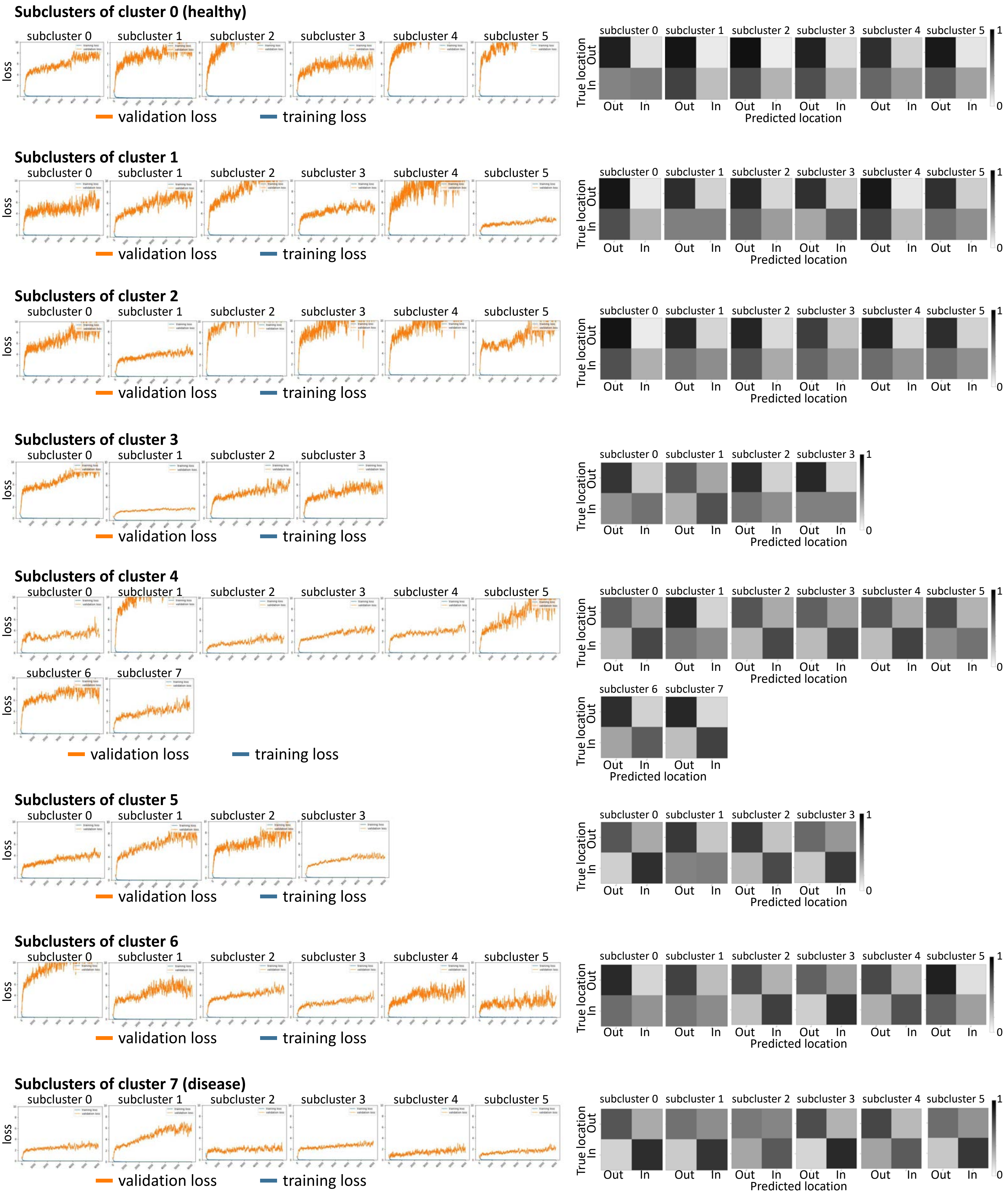

Supplementary Figure 18 c

Proportions of cells inside or outside of breast ducts by subclusters

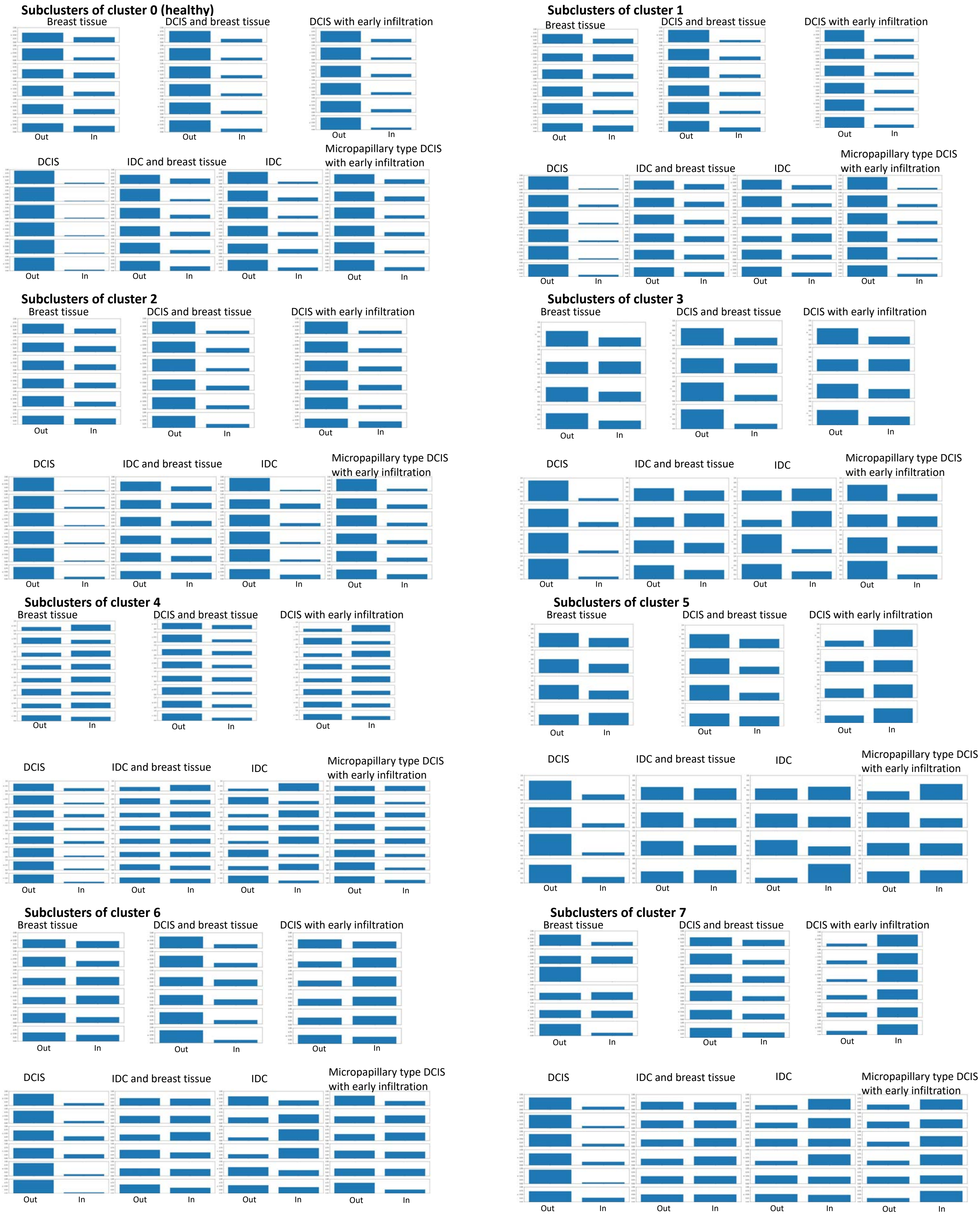

Supplementary Figure 18 d

Supplementary Figure 18. Training curves and confusion matrices of the classifiers trained to distinguish cells inside versus outside of breast ducts based on the VAE latent space embedding or the NMCO features.

(a) The latent representation of a cell computed by the VAE is used as the input to the classifiers.

(b) The same setup as in (a) but the manual segmentation of breast ducts is used instead of the segmentation by thresholding the cytokeratin expression levels.

(c) The NMCO features of a cell are used as the input to the classifiers.

(d) The histograms indicate the proportion of cells inside versus outside of the breast ducts for each phenotypic category within each subcluster.

Distance to duct

Breast tissue

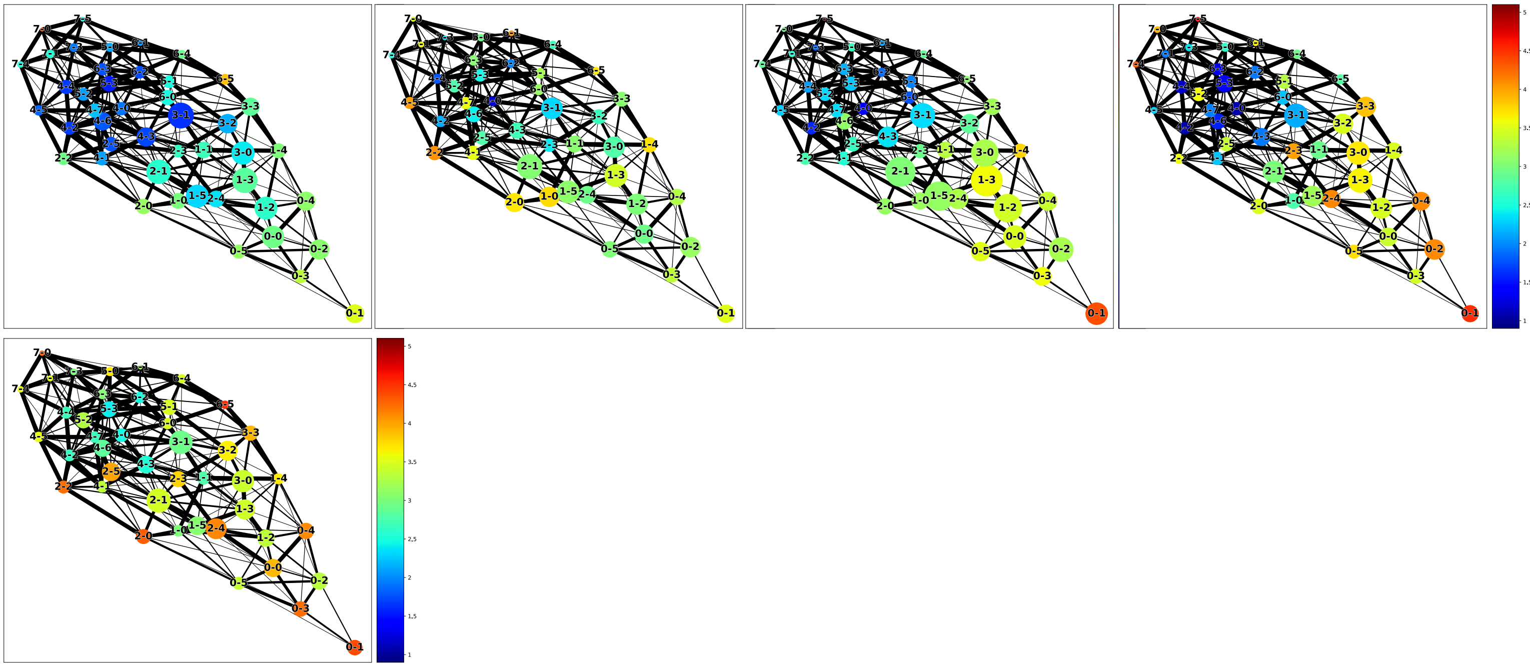

DCIS and breast tissue

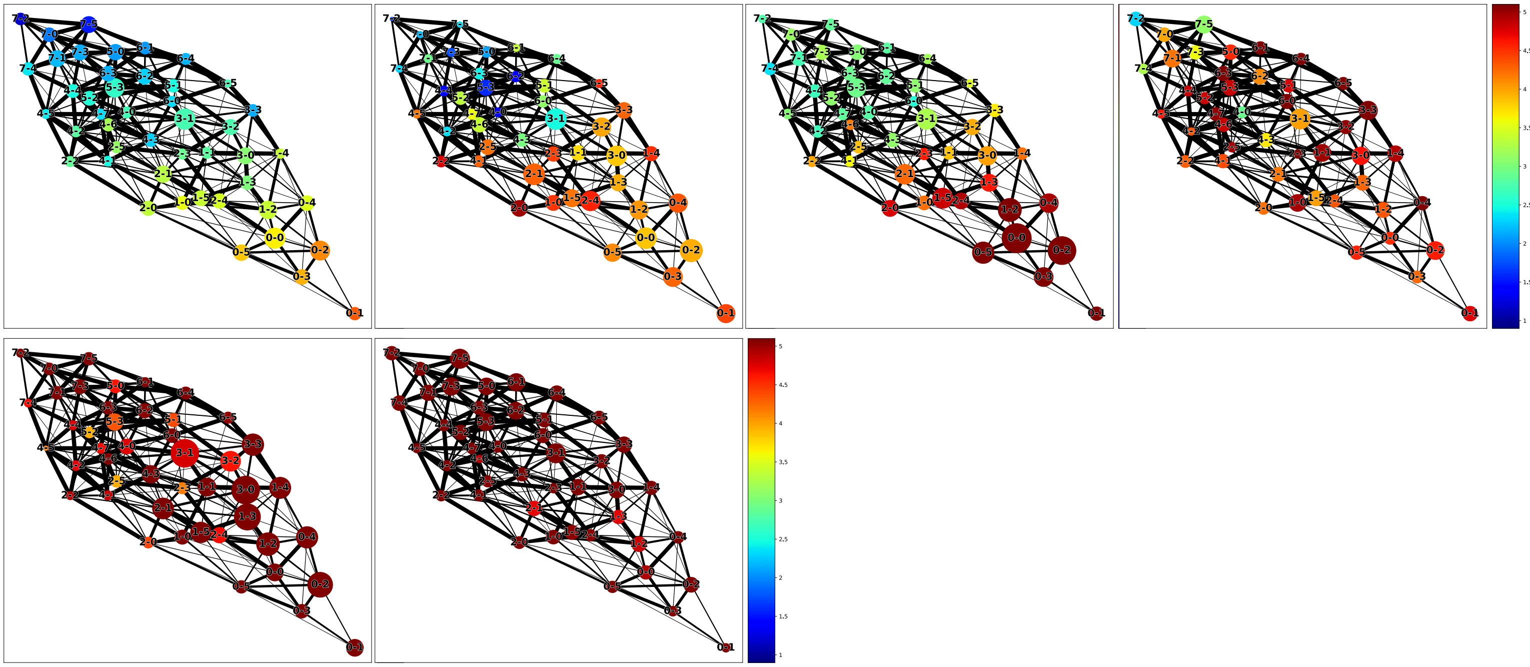

Supplementary Figure 19

Distance to duct

DCIS with early infiltration

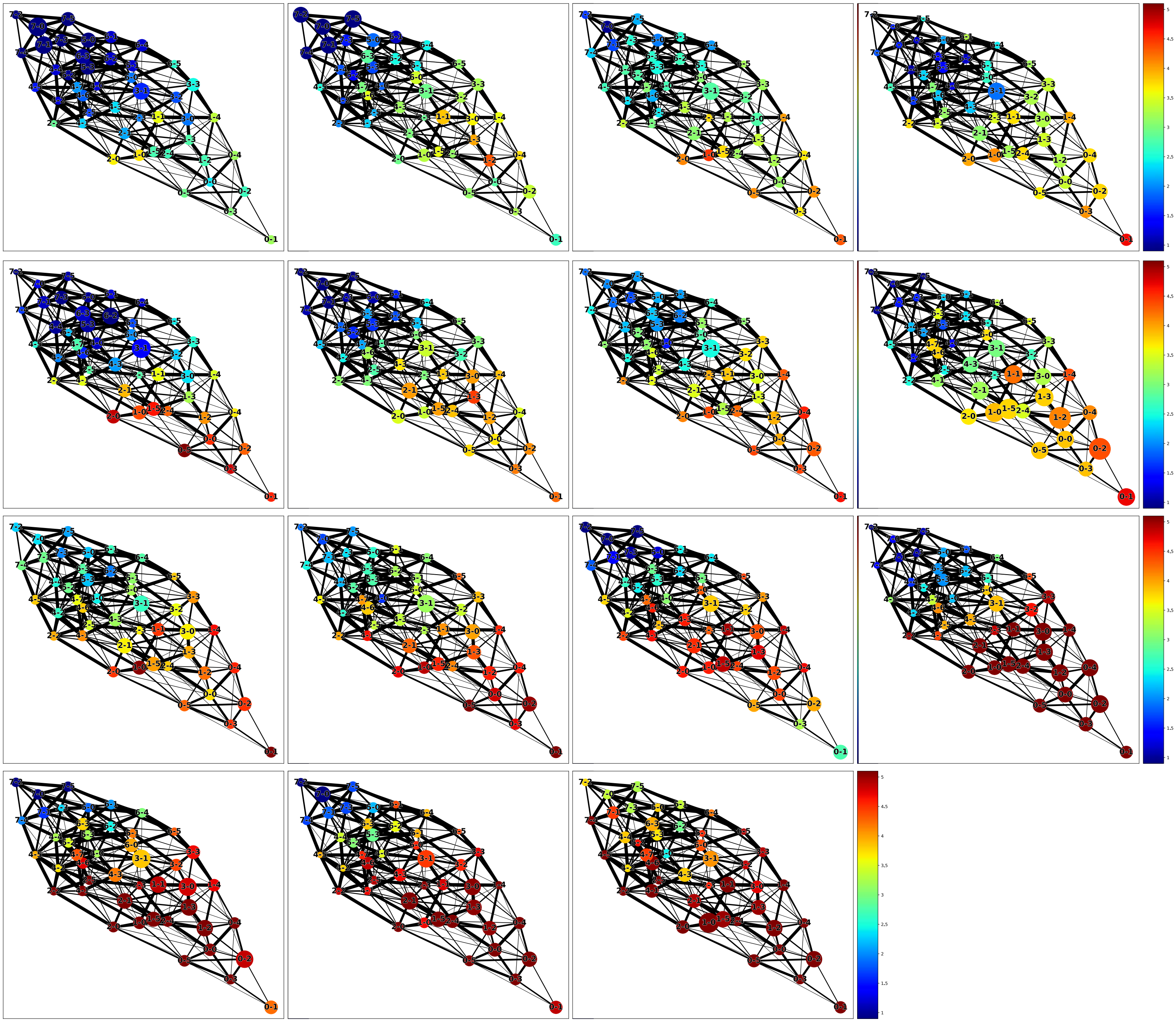

Supplementary Figure 19 (continued)

Supplementary Figure 19. The position of cells relative to breast ducts in each individual sample.
The average distance of a cell to breast ducts is computed for each subcluster and visualized on the PAGA graph. Each plot contains all cells in one sample. Each node represents a subcluster, and its size is proportional to the number of cells in the subcluster.

a Examples of neighborhood images

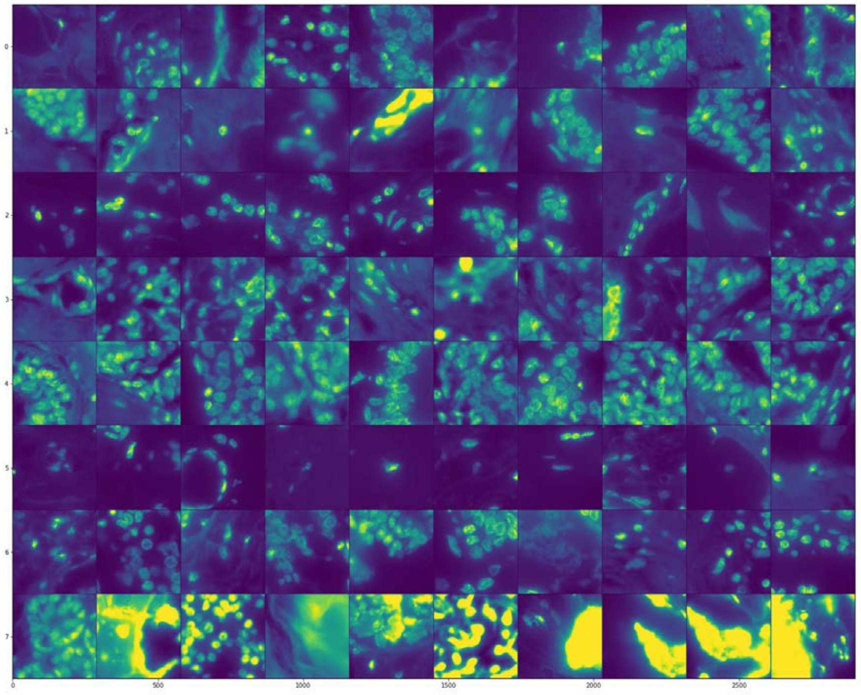

b Cross validation results of pathology classification using different neighborhood sizes to calculate co-localization

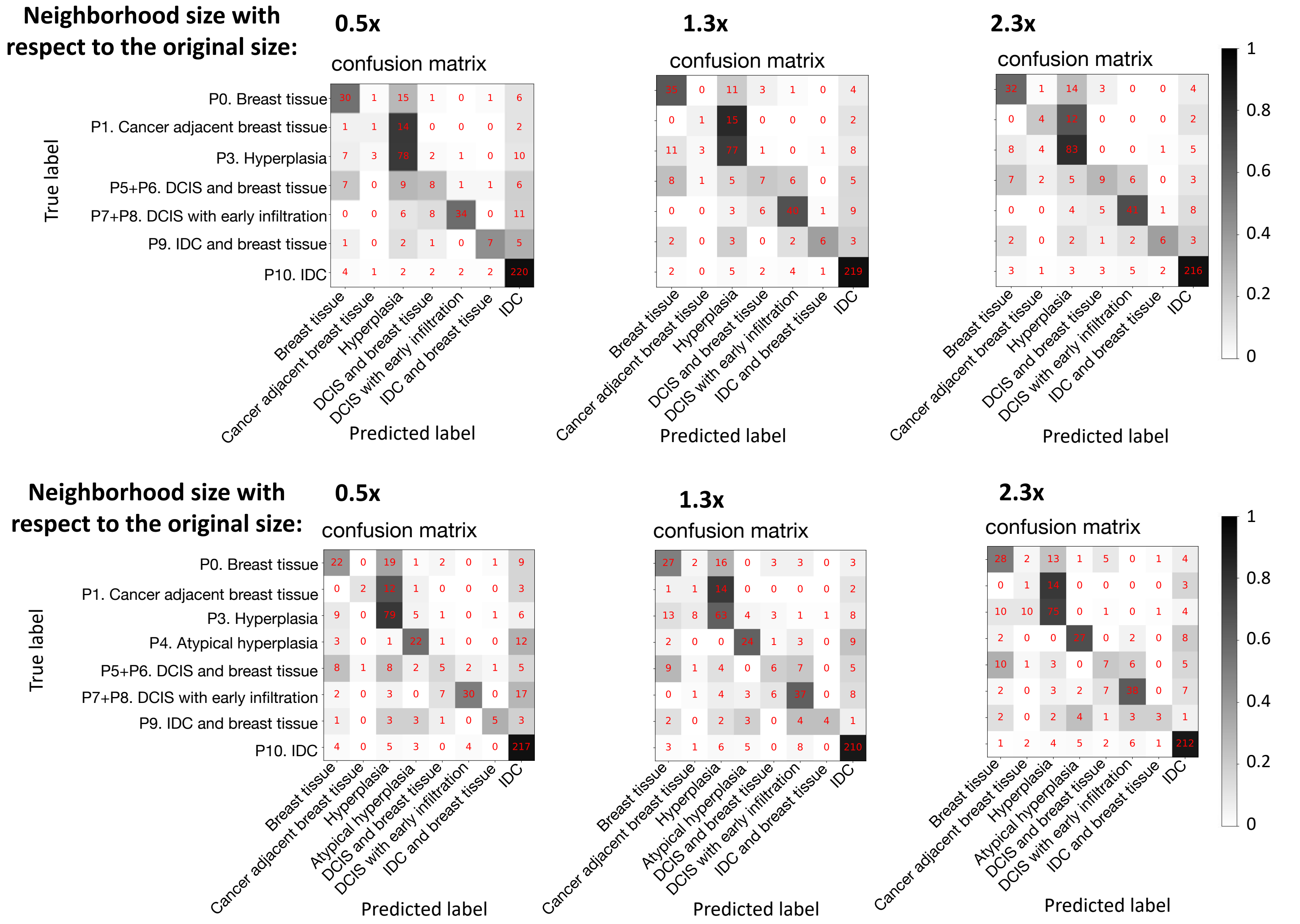

Supplementary Figure 20

Supplementary Figure 20. The predictiveness of the co-localization of cell states in a tissue microarray with respect to phenotypic categories is robust to the choice of the neighborhood size.
(a) Randomly selected examples of neighborhood images with the same size as used in Figure 6.
(b) Confusion matrices as in Figure 6c after retraining the classifiers with different neighborhood sizes using leave-one-patient-out cross validation. The numbers indicate the numbers of samples in each entry. The sizes tested are half, 1.3 times, and 2.3 times the original neighborhood size with a diameter of 51.8 μm. The lower panel contains the classification results trained with the atypical hyperplasia samples, i.e., the samples that might be difficult to distinguish from low-grade DCIS samples.[1,2]

Pathology classification using cell state co-localization, cell state proportions in each sample, or both

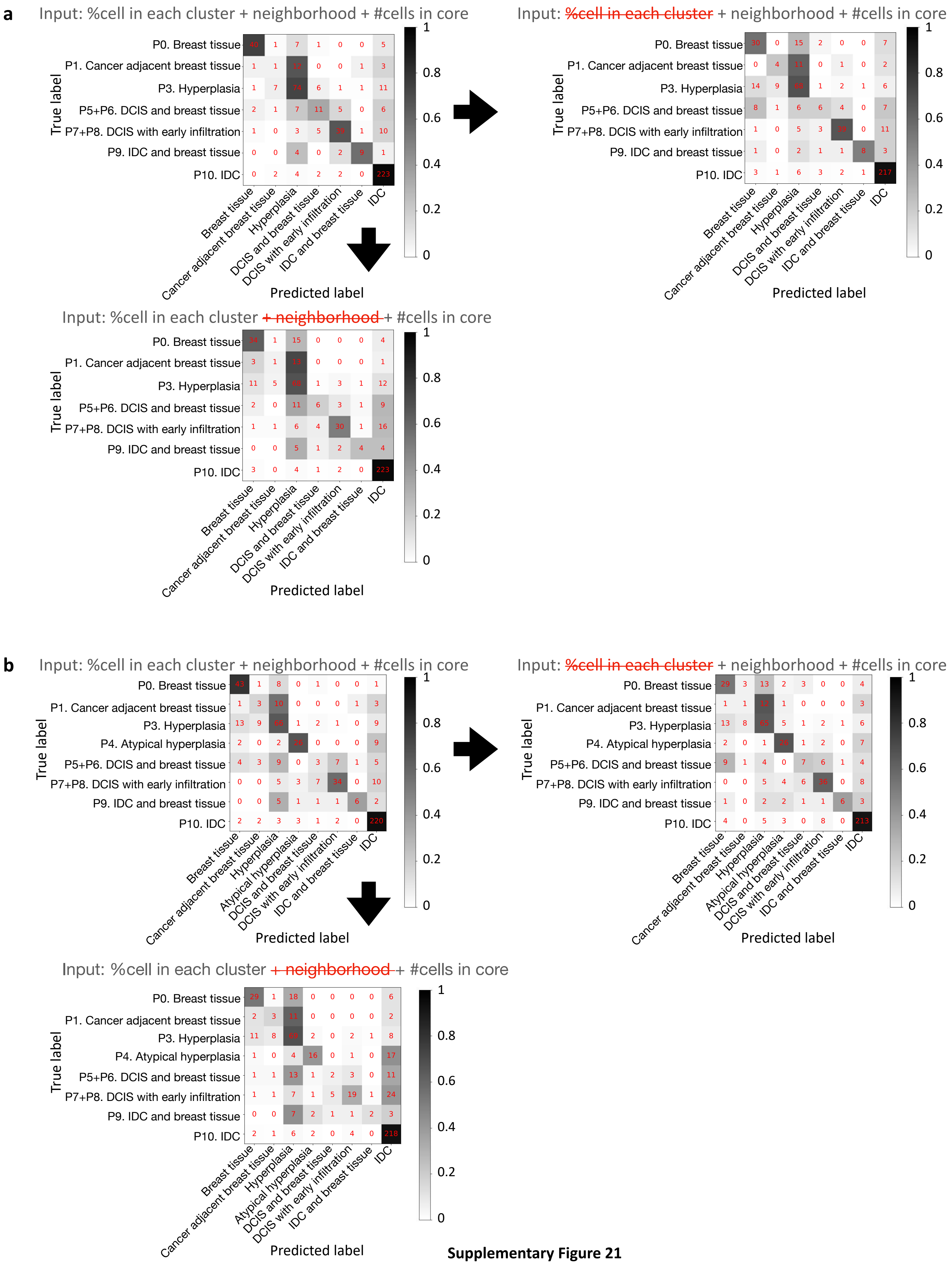

Supplementary Figure 21

Supplementary Figure 21. Cell state co-localization is more predictive of disease phenotypic categories than cell state proportions.

(a) The confusion matrices of three disease-stage classifiers with different inputs were plotted for the leave-one-patient-out cross validation task. The confusion matrices show the fraction of predicted labels for cells sampled from a given phenotypic category. The numbers indicate the numbers of samples in each entry. Top left: Proportions of cells in the eight top-level clusters and in the subclusters are used as input for training the disease-stage classifier described in Figure 6c, in addition to the cell state co-localization matrix. Top right: Results of the classifier without cell cluster proportions are plotted, which is the same plot as in Figure 6c. Bottom: Results of the classifier without cell state co-localization and with only the proportions of cells in the clusters and subclusters as input are plotted.

(b) Same comparison as in (a) for classifiers trained with the atypical hyperplasia samples, i.e., the samples that might be difficult to distinguish from low-grade DCIS samples.[1,2]

Cell state co-localization of misclassified cores vs correctly classified cores - without atypical hyperplasia

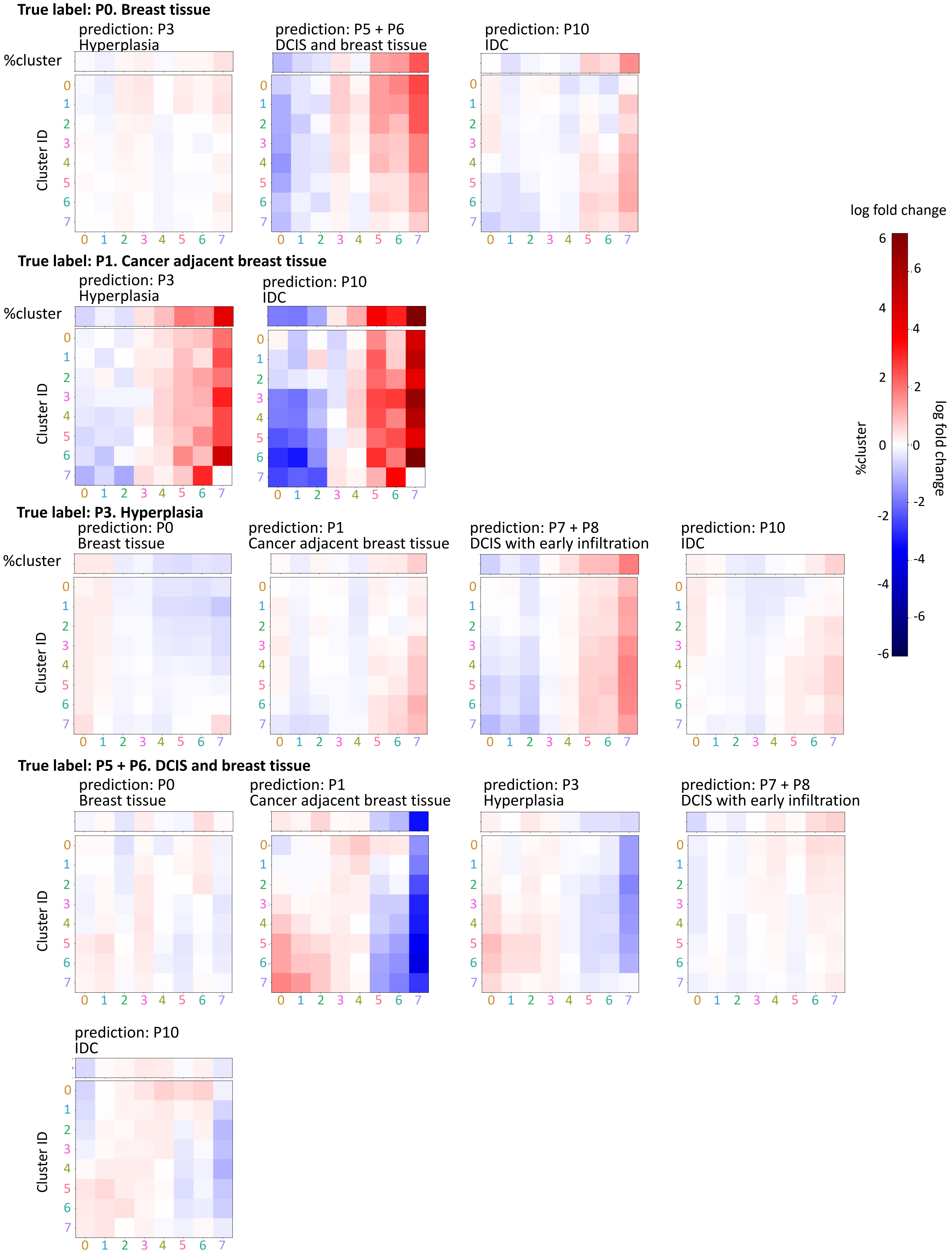

Supplementary Figure 22

Cell state co-localization of misclassified cores vs correctly classified cores - without atypical hyperplasia

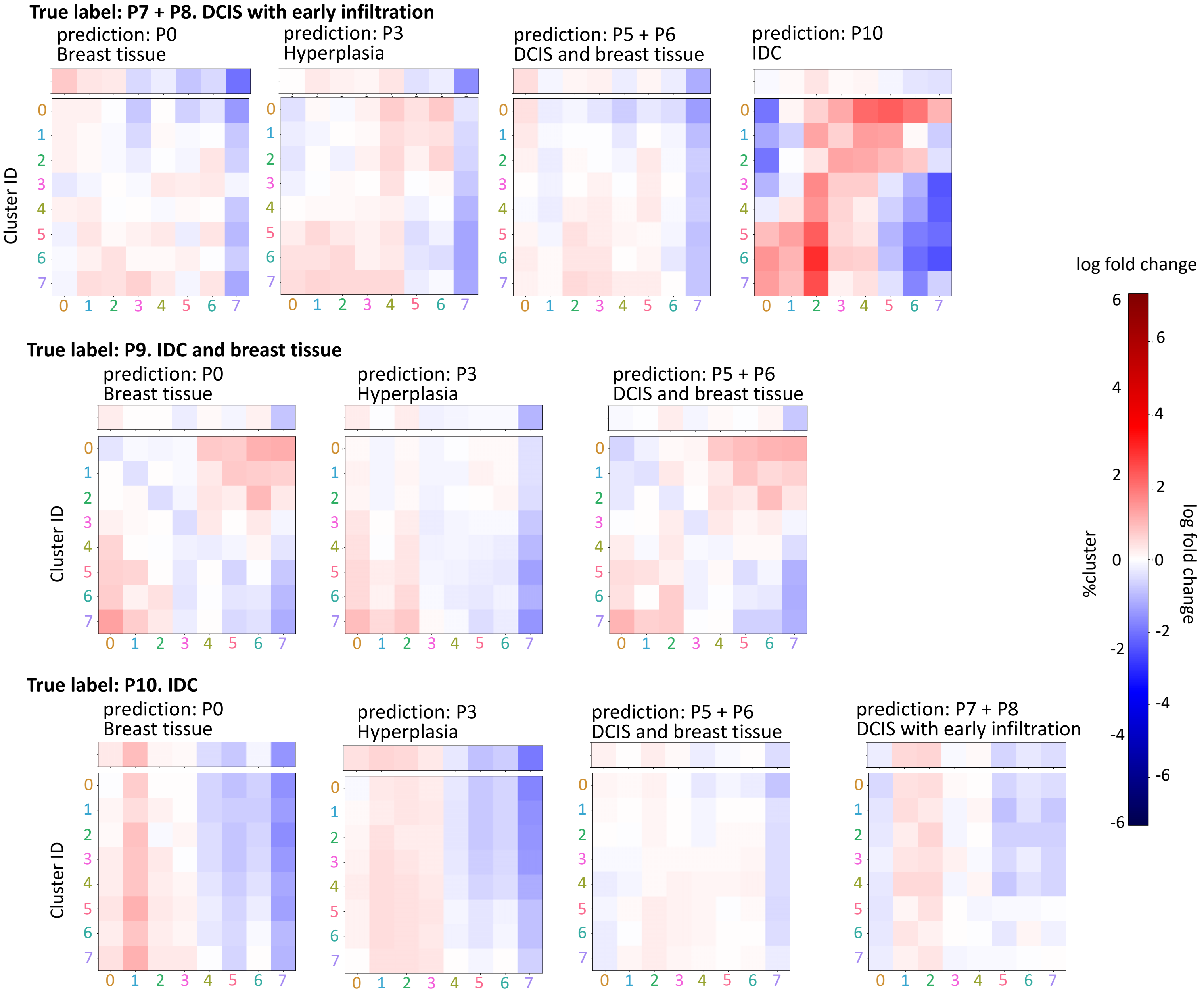

Supplementary Figure 22 (continued)

Supplementary Figure 22. Co-localization patterns of the misclassified samples compared to the correctly classified samples.

The classification is performed using leave-one-patient-out cross validation. The log2 fold changes are plotted. Classification errors were categorized as true phenotypic category of the sample -> predicted phenotypic category of the sample, e.g. Breast tissue -> IDC records the breast tissue samples that were misclassified as IDC. The proportion of cells in each of the eight clusters in the misclassified samples compared to the correctly classified samples are also plotted in terms of the log2 fold change (denoted by %cluster).

Cell state co-localization of misclassified cores vs correctly classified cores - with atypical hyperplasia added

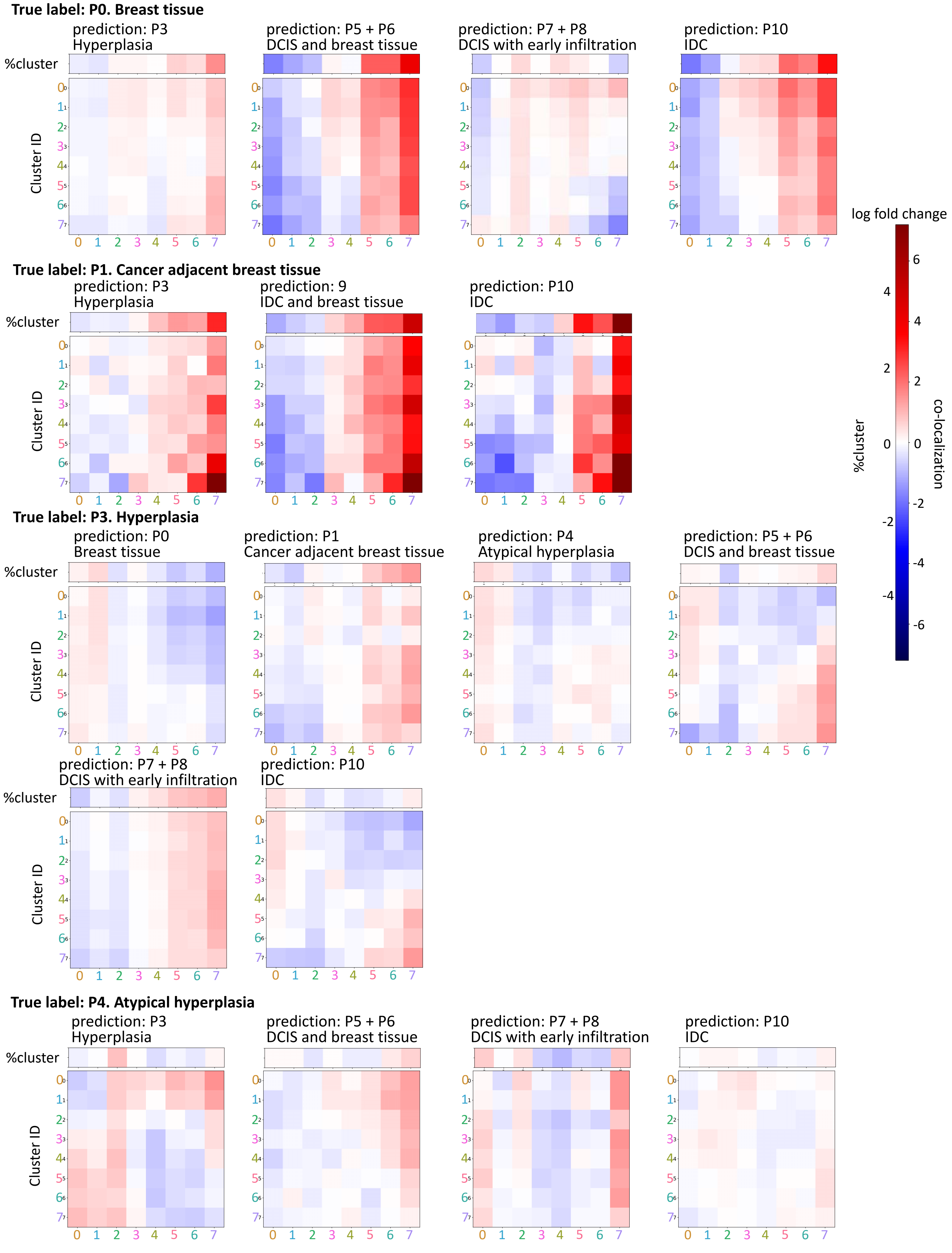

Supplementary Figure 23 a

Cell state co-localization of misclassified cores vs correctly classified cores - - with atypical hyperplasia added

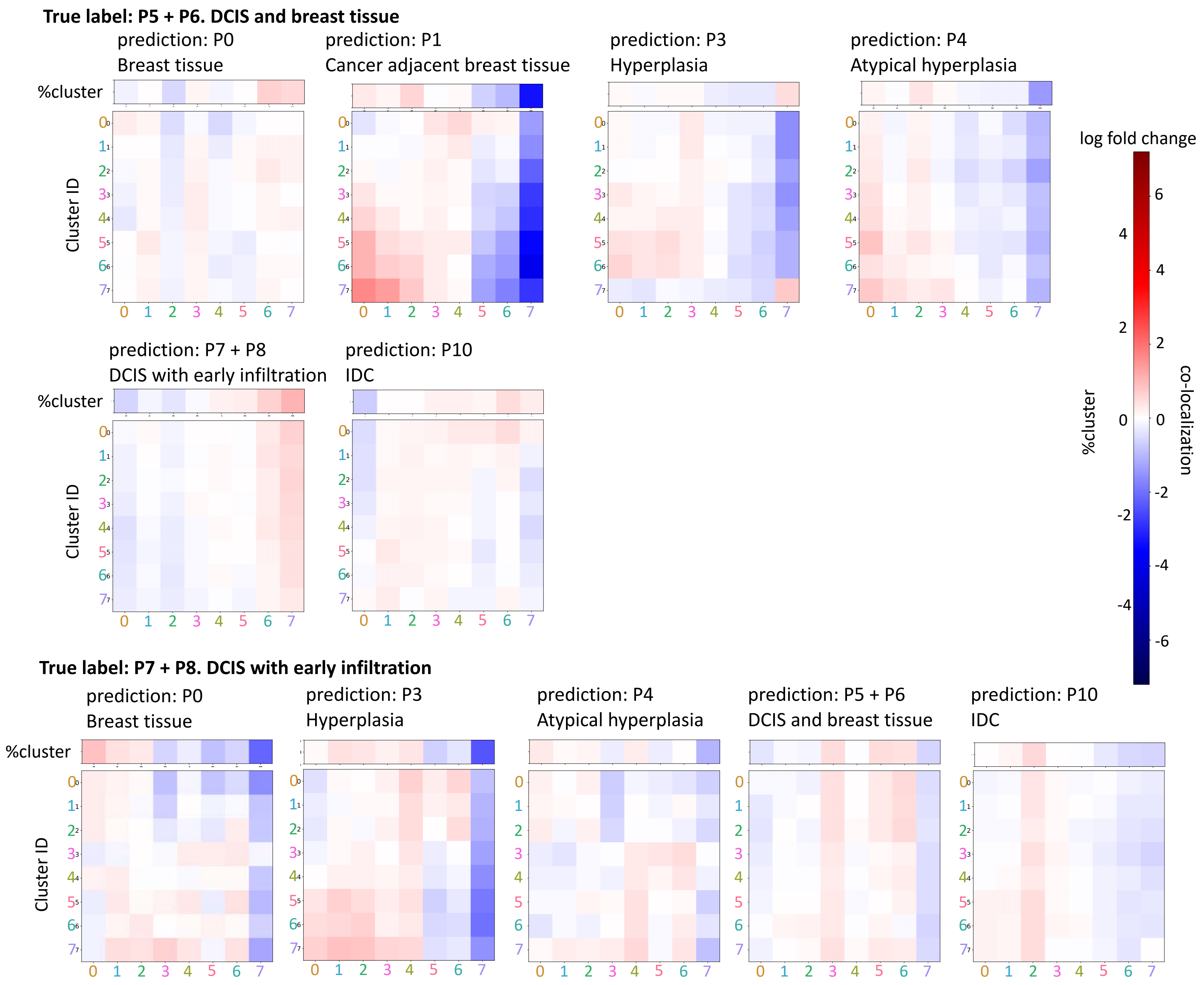

Supplementary Figure 23 b

Cell state co-localization of misclassified cores vs correctly classified cores - with atypical hyperplasia added

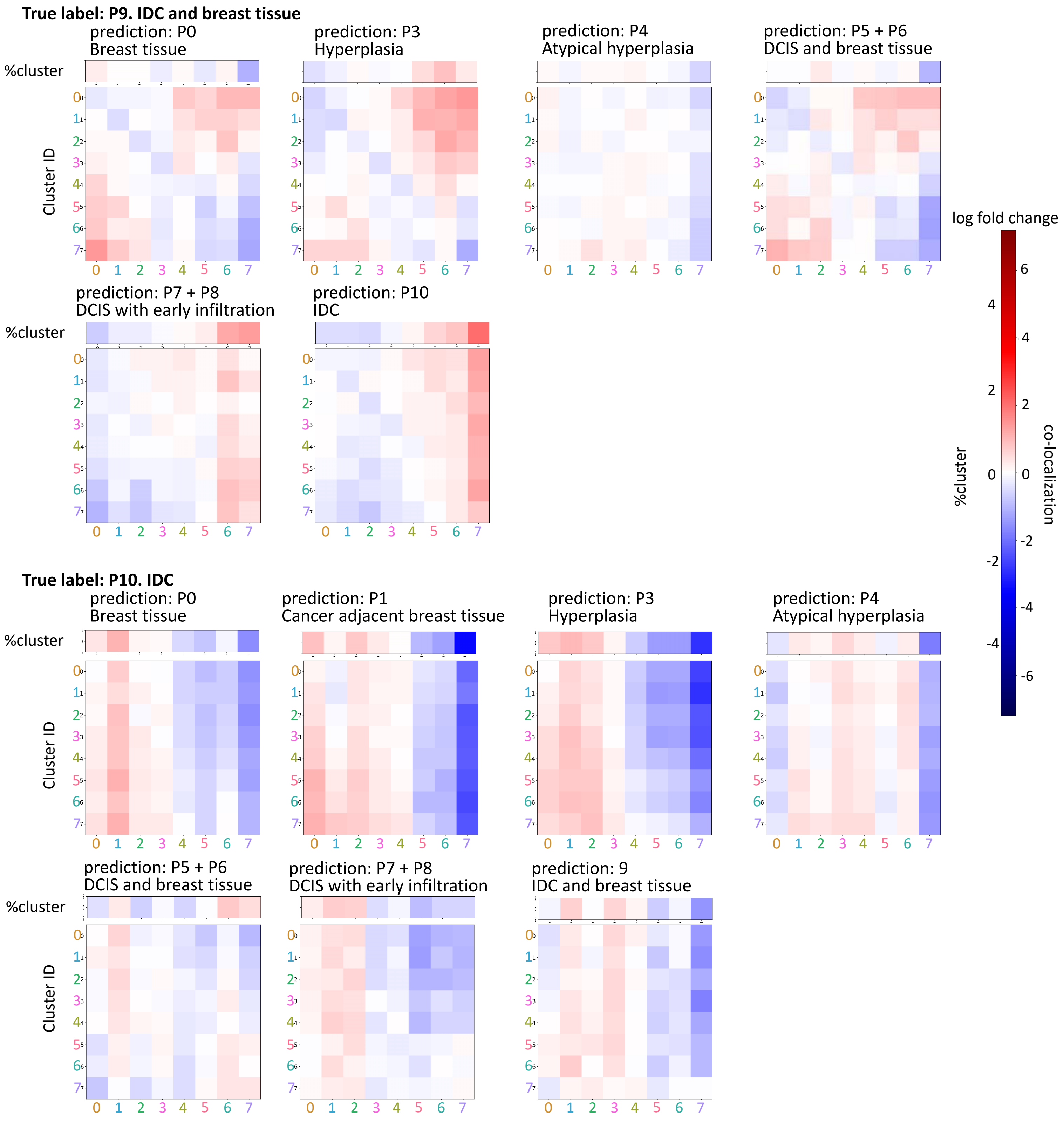

Supplementary Figure 23 c

Supplementary Figure 23. Co-localization patterns of the misclassified samples compared to the correctly classified samples, with Atypical Hyperplasia samples included in the analysis.
The log2 fold changes are plotted. Classification errors were categorized as true phenotypic category of the sample -> predicted phenotypic category of the sample, e.g. Breast tissue -> IDC records the breast tissue samples that were misclassified as IDC. The proportion of cells in each of the eight clusters in the misclassified samples compared to the correctly classified samples are also plotted in terms of the log2 fold change (denoted by %cluster).

a Neural network training losses of three classifiers

**Epoch:**	0	loss_train:	1.3864
**Epoch:**	500	loss_train:	0.1258
**Epoch:**	1000	loss_train:	0.1043
**Epoch:**	1500	loss_train:	0.0863
**Epoch:**	2000	loss_train:	0.0070
**Epoch:**	2500	loss_train:	0.0039
**Epoch:**	3000	loss_train:	0.0144
**Epoch:**	3500	loss_train:	0.0032
**Epoch:**	4000	loss_train:	0.0114
**Epoch:**	4500	loss_train:	0.0237
**Epoch:**	5000	loss_train:	0.0035
**Epoch:**	5500	loss_train:	0.0091
**total**	time:	7.7724s	
**0.0**			
**Epoch:**	0	loss_train:	1.3864
**Epoch:**	500	loss_train:	0.1165
**Epoch:**	1000	loss_train:	0.0986
**Epoch:**	1500	loss_train:	0.0687
**Epoch:**	2000	loss_train:	0.0067
**Epoch:**	2500	loss_train:	0.0073
**Epoch:**	3000	loss_train:	0.0180
**Epoch:**	3500	loss_train:	0.0037
**Epoch:**	4000	loss_train:	0.0089
**Epoch:**	4500	loss_train:	0.0456
**Epoch:**	5000	loss_train:	0.0009
**Epoch:**	5500	loss_train:	0.0059
**total**	time:	7.7667s	
**0.0**			
**Epoch:**	0	loss_train:	1.3864
**Epoch:**	500	loss_train:	0.1147
**Epoch:**	1000	loss_train:	0.0877
**Epoch:**	1500	loss_train:	0.0340
**Epoch:**	2000	loss_train:	0.0101
**Epoch:**	2500	loss_train:	0.0039
**Epoch:**	3000	loss_train:	0.0294
**Epoch:**	3500	loss_train:	0.0047
**Epoch:**	4000	loss_train:	0.0219
**Epoch:**	4500	loss_train:	0.0155
**Epoch:**	5000	loss_train:	0.0010
**Epoch:**	5500	loss_train:	0.0281
**total**	time:	7.7550s	
**0.0**			

b Neural network result

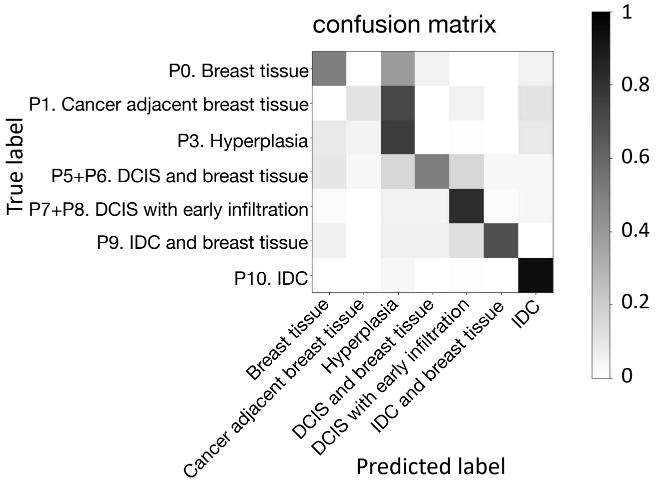

c Logistic regression result

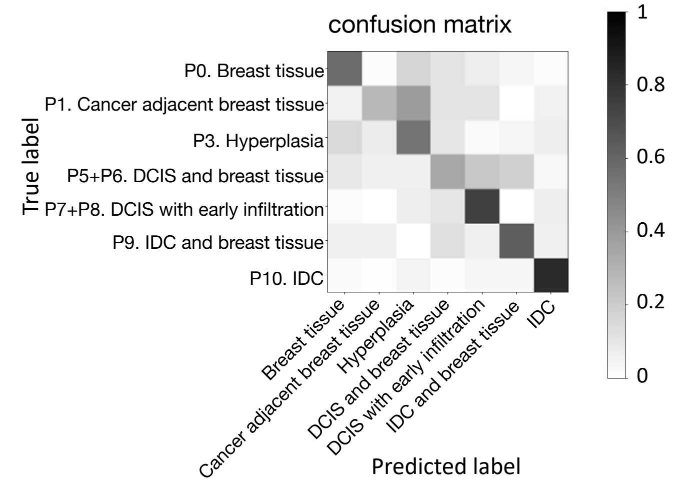

Supplementary Figure 24

Supplementary Figure 24. Training losses and confusion matrices of disease phenotype prediction using neural network and logistic regression.

(a) Examples of training losses in the leave-one-sample-out cross validation tasks are shown for the neural network classifier using co-localization and the total number of cells as input to predict disease phenotypes.

(b) Confusion matrix of the leave-one-out cross validation task in (a).

(c) Confusion matrix of the same prediction task using a logistic regression model.

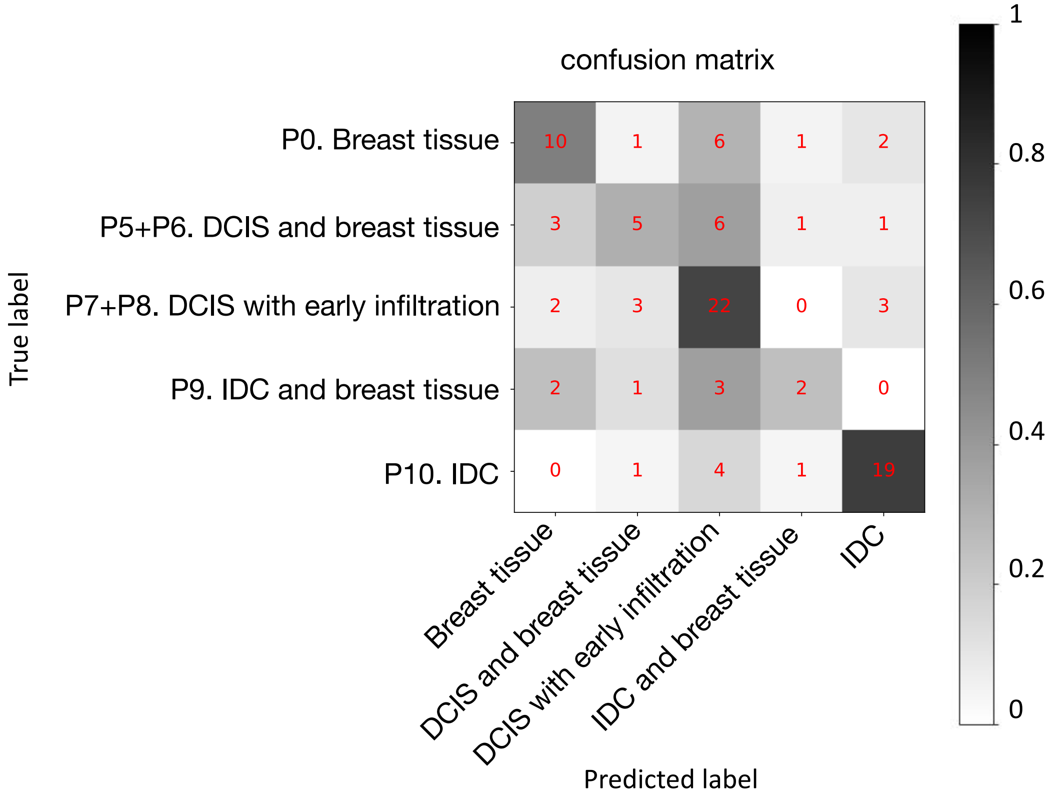

Supplementary Figure 25

Supplementary Figure 25. Confusion matrix of disease phenotype classification using only cells in the ductal region. This shows the results of leave-one-patient-out cross validation and the numbers indicate the numbers of samples in each entry.

Supplementary Data 1. Summary of imaging samples. Each row in the table lists one core used in the experiment; “sample_id” is a unique ID assigned to each core; “slide_id” is the TMA ID, i.e. samples with the same “slide_id” and the same protein stains are on the same TMA; “patient_id” is the patient ID of the core, and cores from the same patient share the same patient ID; “pathology_diagnosis” is the phenotypic category assigned by Biomax. The last four columns indicate the protein stain combinations applied to each core, where the different protein stain combinations of a core are applied to separate samples at different z positions of the core.

Supplementary Data 2. Summary of nuclear morphology and chromatin organization (NMCO) features and the assigned groups by correlation. All NMCO features used in our analysis are listed with a description of what each feature measures. The “Group” column lists which feature group each NMCO feature is assigned to based on correlation (Methods). “Ungrouped” means that the feature is not strongly correlated with any other features, i.e., correlation is equal to or less than 0.8.

Supplementary References

1. Tozbikian, G. *et al.* Atypical Ductal Hyperplasia Bordering on Ductal Carcinoma In Situ: Interobserver Variability and Outcomes in 105 Cases. *Int. J. Surg. Pathol.* **25**, 100–107 (2017).
2. Pinder, S. E. & Ellis, I. O. The diagnosis and management of pre-invasive breast disease: Ductal carcinoma in situ (DCIS) and atypical ductal hyperplasia (ADH) – current definitions and classification. *Breast Cancer Res.* **5**, 254 (2003).
