# Supplementary material for: Unsupervised representation learning of chromatin images identifies changes in cell state and tissue organization in DCIS
Source: Nat Commun. 2024 Jul 20;15:6112. doi: 10.1038/s41467-024-50285-1 (PMC11271523; doi:10.1038/s41467-024-50285-1)
Supplement: Supplementary file 3 — Description of Additional Supplementary Files [file 41467_2024_50285_MOESM3_ESM.pdf]

Supplementary Data 1.
Summary of imaging samples. Each row in the table lists one core used in the experiment; “sample_id” is a unique ID assigned to each core; “slide_id” is the TMA ID, i.e. samples with the same “slide_id” and the same protein stains are on the same TMA; “patient_id” is the patient ID of the core, and cores from the same patient share the same patient ID; “pathology_diagnosis” is the phenotypic category assigned by Biomax. The last four columns indicate the protein stain combinations applied to each core, where the different protein stain combinations of a core are applied to separate samples at different z positions of the core.

Supplementary Data 2.
Summary of nuclear morphology and chromatin organization (NMCO) features and the assigned groups by correlation. All NMCO features used in our analysis are listed with a description of what each feature measures. The “Group” column lists which feature group each NMCO feature is assigned to based on correlation (Methods). “Ungrouped” means that the feature is not strongly correlated with any other features, i.e., correlation is equal to or less than 0.8
